# The Effect of Larval Exposure to Heavy Metals on the Gut Microbiota Composition of Adult *Anopheles arabiensis* (Diptera: Culicidae)

**DOI:** 10.3390/tropicalmed9100249

**Published:** 2024-10-21

**Authors:** Ashmika Singh, Shristi Misser, Mushal Allam, Wai-Yin Chan, Arshad Ismail, Givemore Munhenga, Shüné V. Oliver

**Affiliations:** 1 Wits Research Institute for Malaria, School of Pathology, Faculty of Health Sciences, University of the Witwatersrand, Johannesburg 2193, South Africa; 2 Centre for Emerging Zoonotic and Parasitic Diseases, National Institute for Communicable Diseases, Division of the National Health Laboratory Service, Johannesburg 2193, South Africa; 3 Department of Genetics and Genomics, College of Medicine and Health Sciences, United Arab Emirates University, Abu Dhabi 15551, United Arab Emirates; 4 Antimicrobial Research Unit, School of Health Sciences, University of KwaZulu-Natal, Durban 4041, South Africa; 5 Department of Biochemistry, Genetics and Microbiology (BGM), Forestry and Agricultural Biotechnology Institute (FABI), University of Pretoria, Pretoria 0028, South Africa; annie.chan@witsdih.ac.za; 6 Sequencing Core Facility, National Institute for Communicable Diseases, Division of the National Health Laboratory Service, Johannesburg 2193, South Africa; arshadi@nicd.ac.za; 7 Department of Biochemistry and Microbiology, Faculty of Science, Engineering and Agriculture, University of Venda, Thohoyandou 0950, South Africa; 8 Institute for Water and Wastewater Technology, Durban University of Technology, Durban 4000, South Africa

**Keywords:** paratransgenesis, pollution, 16S rRNA, malaria, copper, cadmium

## Abstract

*Anopheles arabiensis* is a highly adaptable member of the *An*. *gambiae* complex. Its flexible resting behaviour and diverse feeding habits make conventional vector control methods less effective in controlling this species. Another emerging challenge is its adaptation to breeding in polluted water, which impacts various life history traits relevant to epidemiology. The gut microbiota of mosquitoes play a crucial role in their life history, and the larval environment significantly influences the composition of this bacterial community. Consequently, adaptation to polluted breeding sites may alter the gut microbiota of adult mosquitoes. This study aimed to examine how larval exposure to metal pollution affects the gut microbial dynamics of *An. arabiensis* adults. Larvae of *An. arabiensis* were exposed to either cadmium chloride or copper nitrate, with larvae reared in untreated water serving as a control. Two laboratory strains (SENN: insecticide unselected, SENN-DDT: insecticide selected) and F_1_ larvae sourced from KwaZulu-Natal, South Africa, were exposed. The gut microbiota of the adults were sequenced using the Illumina Next Generation Sequencing platform and compared. Larval metal exposure affected alpha diversity, with a more marked difference in beta diversity. There was evidence of core microbiota shared between the untreated and metal-treated groups. Bacterial genera associated with metal tolerance were more prevalent in the metal-treated groups. Although larval metal exposure led to an increase in pesticide-degrading bacterial genera in the laboratory strains, this effect was not observed in the F_1_ population. In the F_1_ population, *Plasmodium*-protective bacterial genera were more abundant in the untreated group compared to the metal-treated group. This study therefore highlights the importance of considering the larval environment when searching for local bacterial symbionts for paratransgenesis interventions.

## 1. Introduction

South Africa is part of the “Elimination 8”, a grouping of eight countries in southern Africa aiming to eliminate malaria [1]. The elimination of malaria in South Africa is hindered by various factors, including the complex composition of malaria vectors. All the species involved in disease transmission in South Africa are known to bite and rest outdoors. As a result, conventional chemical interventions, such as indoor residual spraying and long-lasting insecticide-treated nets are less effective since they primarily target mosquitoes that bite and rest indoors [2]. Consequently, the presence of outdoor-biting and outdoor-resting mosquito populations contributes to ongoing low-level transmission of the disease outdoors, referred to as residual malaria [3,4]. This is a particular challenge for South Africa’s elimination agenda.

Although current chemical interventions remain effective in South Africa, they are not efficient against outdoor biting vectors. Additionally, insecticide resistance in local mosquito populations presents further challenges to these interventions [5]. Therefore, alternative and supplementary vector control tools are needed to achieve the goal of malaria elimination. Ideally, these should be a non-chemical intervention. One potential option is transmission blocking, possibly through genetic modification. A promising approach is paratransgenesis, a form of biocontrol where microbial symbionts are used to induce refractoriness in vectors [6,7]. 

Paratransgenesis is based on the manipulation of symbionts, usually bacteria, due to their large influence on the life history of the mosquito. The gut microbiota in particular play a crucial role during the mosquito developmental cycle. The gut microbiota are essential for larval development [8], with the correct bacterial composition needed for the larvae to pupate [9]. The gut microbiota are also crucial during digestion, particularly blood digestion [10]. The bacterial composition is also implicated in insecticide resistance [11]. The most important factor for paratransgenesis, however, is that gut microbiota are a critical immunomodulator and influence the escape of *Plasmodium* parasites from the gut [12]. The midgut is a major barrier for parasite escape, and if contained in the gut, the chances of transmission blocking are significantly increased [13]. Gut microbiota modulate the formation of the peritrophic matrix, a chitinous membrane that surrounds the ingested blood meal [14]. This protects the mosquito from a range of ingested pathogens, including the *Plasmodium* parasite [15]. Microbial components also modulate the immune system to eliminate the parasite [13]. There are a range of bacterial species that have been identified as potential paratransgenesis candidates. These symbionts are suggested for the control of a range of vector-borne diseases [16,17]. 

Many paratransgenesis candidates are based on their natural capacity to inhibit *Plasmodium* parasites. Other species are candidates because of their association with refractoriness and would require genetic modification to improve their anti-*Plasmodium* function [7]. However, not all paratransgenesis candidates are genetically modified. As an example, the *Enterobacter* Esp_Z strain identified from Zambian *An. arabiensis* is a naturally occurring symbiont capable of inhibiting parasite development [18]. The microsporidian Microsporidia MB is capable of inhibiting parasite development and was also discovered in *An. arabiensis* [19]. The most used paratransgenesis candidate is the intracellular bacterium *Wolbachia*. The most effective use of the symbiont has been where local strains have been used [20]. As such, successful paratransgenesis efforts require an intensive understanding of the local symbionts where the intervention is to take place. 

As mining local populations for potential symbionts is important for this intervention, it is important to understand the importance of bacterial dynamics. It has been recognised that bacterial dynamics differ by season [21]. Furthermore, it has been demonstrated that the larval environment plays a critical role in adult bacterial composition [22]. As such, when looking for local symbionts, it is important to understand the effect of the environment on bacterial composition. 

*Anopheles arabiensis* is a member of the *An. gambiae* complex implicated in malaria transmission in South Africa [23]. This species is generally difficult to control [24] but notably, it has been reported to be adapting to breeding in polluted areas [25]. This is significant as the *An. gambiae* complex typically breeds in clean, clear, sunlit bodies of water [26]. This has implications for the expansion of the range of this species, but critically, this adaptation has a range of effects on mosquito life history. This includes the selection of insecticide resistance [27]. As such, adaptation to pollution is important to consider when examining the larval environment’s effect on bacterial composition.

In this study, the effect of larval exposure to heavy metals on bacterial composition and diversity was examined on two laboratory strains of *An. arabiensis*. The first is the insecticide unselected primarily insecticide susceptible strain named SENN, and the insecticide selected strain named SENN-DDT. The use of both selected and unselected strains is because it has been previously demonstrated that insecticide resistance affects the gut bacterial composition [28]. 

The two laboratory strains in this study have previously been used to examine the effect of larval exposure to heavy metals. *Anopheles arabiensis*, like *An. gambiae*, has been adapting to breeding in polluted sites, especially metal-polluted water [29]. The first example of *An. gambiae* breeding in polluted water highlighted that the levels of lead, copper, and cadmium in particular were high in the breeding site [30]. As such, these metals have been the focus of metal adaption studies in the complex [31,32,33]. Copper and cadmium have been demonstrated to have effects on the life history of the SENN and SENN-DDT strains [34]. This effect lasts to the second generation, even if the adults breed in clean water [35]. There have also been demonstrations of the effect of these metals on the epigenetic architecture of these strains [36]. This study therefore expands the study of the effect of pollutants on these two *An. arabiensis* strains by examining the effect of these stressors on the gut microbiota. This will be contextualised by a comparison to wild mosquitoes. 

## 2. Materials and Methods

This study compared the dynamics of the effect of larval metal exposure of two laboratory strains of *An. arabiensis* with that of F_1_ *An. arabiensis* from KwaZulu-Natal, South Africa as a wild-type comparator. This will offer insights into how insecticide-resistant phenotypes influence this dynamic. Additionally, it will demonstrate the value of using laboratory strains to understand the environmental impact on bacterial dynamics. 

All mosquitoes used in this study were housed in the Botha de Meillon (BDMI) insectary in Sandringham, Johannesburg. Mosquito husbandry procedures were performed as per [37]. In brief, mosquitoes were reared at 25 °C (±2 °C) at a humidity of 80% (±5%). Larvae were reared on a diet of Beano™ dog biscuits and brewer’s yeast mixed at a ratio of 3:1, respectively. Adults were maintained with a 12:12 h light cycle with a 30-min dawn/dusk cycle. Adults were maintained with ad libitum access to 10% sucrose. 

Two laboratory strains of *An. arabiensis* were used. SENN is an unselected susceptible strain colonised in 1980 and originates from Gezira, Sudan. This strain had incipient DDT and malathion resistance, which has subsequently declined to near susceptibility [38]. From this strain, a resistant strain was selected. The SENN-DDT strain is an insecticide-resistant strain selected by continuous exposure to 4% DDT. The selection started in 1995 and continues until the present. SENN-DDT displays resistance to DDT, deltamethrin, λ-cyhalothrin, and malathion [39]. The resistance in this strain is mediated by elevated enzyme activity of cytochrome P450s, general esterases, and glutathione S-transferases [40]. 

The F_1_ adults were obtained from adults collected from Mamfene, KwaZulu-Natal, South Africa (S27°20′17.95″; E32°12′53″). Collections took place between November 2019 and April 2020 [5]. Adults were collected from clay pots and modified bucket traps [5]. Once transported from the field site to the BDMI, they were kept in quarantine and not allowed any additional blood meals. Females were individually tubed to allow for the generation of isofamily lines. Upon laying a batch of eggs, the females that laid eggs were placed on silica and identified morphologically according to [41]. Members of the *An. gambiae* complex were identified by conventional polymerase chain reaction (PCR) according to the method of [42]. If identified as *An. arabiensis*, samples were pooled and reared according to the standard insectary procedures described previously. Adults used for F_1_ screening represent offspring from 50 families. Metal exposures were performed as per [34]. Samples were exposed to the maximum acceptable toxicity concentration (MATC), as it represented the lowest possible concentration that would qualify as pollution. In brief, for the laboratory strains, the 100 first instar larvae less than 24 h old were placed in 1000 mL water. To this a metal solution was added to a final concentration of either 0.36 μg/L for cadmium chloride or 1.86 μg/L for copper nitrate [32]. Larvae reared in untreated water served as a control. For the F1 larvae, 50 larvae per 500 mL were pooled at the same concentrations as for the laboratory strains. These experiments were replicated three times. All larvae were fed a set amount of food (1 mg food per larvae, twice daily). To collect the specimens for sequencing, the adults were collected and kept in cages corresponding to their emergence date. Adults were cold killed at the age of three days. During the period prior to their killing, females were not allowed a blood meal. These mosquitoes were used for subsequent DNA extractions. 

### 2.1. Preparation of Samples for Sequencing

The external surface of the samples was sterilised using 70% ethanol. The midguts were resected in a sterile manner at 40× magnification using an Olympus SZ40 (Olympus LS, Tokyo, Japan). Three guts were used per replicate, with five replicates prepared for each treatment. Total genomic DNA was extracted using the Qiagen blood and tissue kit (Qiagen: 69506, Hilden, North Rhine-Westphalia, Germany). The optional RNAse-A treatment was performed using the kit enzyme (Catalogue number: 19101). An extraction control was included in the reaction (Zymo Research: D6300, Irvine, CA, USA). 

The V3–V4 hypervariable region of the bacterial 16S rRNA gene was amplified using conventional PCR [43]. The amplification was performed according to the Illumina 16S Metagenomic Sequencing Library Preparation protocol (Illumina TM, San Diego, CA, USA). Samples were amplified using the Kappa Hi-Fi HotStart ReadyMix (KAPA Biosystems: KK2601, Cape Town, South Africa). Primers for amplification were purchased from Integrated DNA Technologies (Coralville, IA, USA). The sequences for the primers were as follows: 5′ GTC TCG TGG GCT CGG AGA TGT GTA TAA GAG ACA GGA CTA CHV GGG TAT CTA ATC C 3′ and reverse primer 5′ TCG TCG GCA GCG TCA GAT GTG TAT AAG AGA CAG CCT ACG GGN GGC WGC AG 3′ (4 nmol Ultramer^®^ DNA Oligo 55 bases: Integrated DNA Technologies, Coralville, IA, USA). The extraction control described above was included in the amplification, as was an amplification control (Zymo Research: D6306, Irvine, CA, USA).

Library preparation was performed according to the standard instructions of the 16S Metagenomic Sequencing Library Preparation protocol (Illumina TM, San Diego, CA, USA). This included PCR cleanup, Nextera index PCR, and quality control in the form of validation using the 4200 TapeStation (Agilent Technologies, Santa Clara, CA, USA). The library was quantified, normalised, pooled, and denatured. Libraries were then sequenced on the MiSeq platform using the MiSeq Reagent kit v3 (Illumina, San Diego, CA, USA) and paired-end 2 × 300 bp sequencing was performed at the Sequencing Core Facility, National Institute for Communicable Diseases. The buffer-only negative control was also amplified and sequenced.

### 2.2. Bioinformatics

TrimGalore (v0.6.5-1) was used to filter and pair-end trim sequences to remove the Nextera adapter sequences. To ensure clean data were used for downstream analysis, quality control was performed using MultiQC (v1.6). All the downstream analyses were performed in Rstudio (v4.2.1), including classification, abundance estimations, statistical analysis, and visualisation. The Dada2 package (v1.24.0) was used for quality inspection, filtering, trimming, dereplication, sample inference, merging paired-end reads, and removal of chimeric sequences [44]. Amplicon sequence variants (ASVs) and the ASV abundance estimates were determined using training sequence sets based on the SILVA reference database v138; https://zenodo.org/record/4587955#.Y9JGXnZBxPY (accessed on 13 March 2024). Dada2 outputs were then constructed into phyloseq objects using the phyloseq package (v1.40.0) [45]. The phyloseq objects were then used for further analyses. 

Alpha diversity is a measure of the diversity within a sample. This can be measured as the number of species present (species richness) or the abundance and distribution of species (species diversity). 

For alpha diversity, species richness was determined using the Chao1 and ACE index while diversity was measured using the Shannon–Weiner and Simpson dominance index. The Wilcoxon rank-sum tests were used to compare alpha diversity between strains (or treatments). 

Beta diversity is a difference in diversity between samples. For beta diversity, ordination plots were constructed using the non-metric multidimensional scaling (NMDS) method. The data clustering between the different strains for each metal treatment for the NMDS plot was statistically assessed using a PERMANOVA (permutation test with pseudo-F ratios) as implemented in the adonis function in the vegan package https://github.com/vegandevs/vegan (accessed on 13 March 2024). To visualise overlapping microbial communities between the different families, genera and species, UpSet plots were generated using Venn Diagram (v1.7.3) and UpsetR (v1.4.0) [46]. Differential abundance analysis between sample groups was performed using DESeq2 (v1.24.0) [47]. Except for the UpSet plots, all plots were constructed using ggplot2 (v3.4.0) [48].

## 3. Results

### 3.1. Alpha Diversity

**Figure 1:**
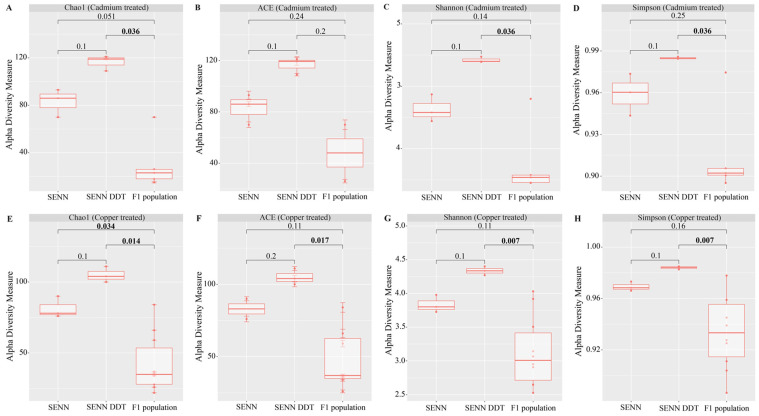
Comparison of alpha diversity of laboratory and F_1_ population *An. arabiensis* adults exposed to metal pollutants as larvae. (**A**) Chao1 index, a measure of species richness, after cadmium treatment. (**B**) ACE index, a measure of species richness, after cadmium treatment. (**C**) Shannon–Weiner index, a measure of species diversity. (**D**) Simpson dominance index, a measure of species diversity, after cadmium treatment. (**E**) Chao1 index, a measure of species richness, after copper treatment. (**F**) ACE index, a measure of species richness, after copper treatment. (**G**) Shannon–Weiner index, a measure of species diversity, after copper treatment. (**H**) Simpson dominance index, a measure of species diversity, after copper treatment. Values indicated in the figure are *p*-values, with significant values in bold.

A summary of all ASVs identified in the study is provided as Supplementary Table S1. There was no significant difference in alpha diversity between SENN and SENN-DDT after cadmium treatment. Except for the ACE index (*p* = 0.2), the F_1_ population had a significantly lower richness and diversity than that of SENN-DDT (Chao 1: *p* = 0.036, Shannon–Weiner: *p* = 0.036, Simpson *p* = 0.036) (Figure 1A–D). 

There was no significant difference in alpha diversity between SENN and SENN-DDT after copper treatment. In all indices, the F_1_ population had significantly lower indices compared to SENN-DDT (Chao1: *p* = 0.014, ACE: *p* = 0.017, Shannon: *p* = 0.007, Simpson: *p* = 0.007) (Figure 1E,F). The F_1_ population had a lower Chao1 index compared to SENN (*p* = 0.034) in addition to that of SENN-DDT (Figure 1A–D). 

There was no significant difference in the combined alpha diversity indices of all three strains when comparing the untreated, cadmium, and copper treatments. 

### 3.2. Beta Diversity

**Figure 2:**
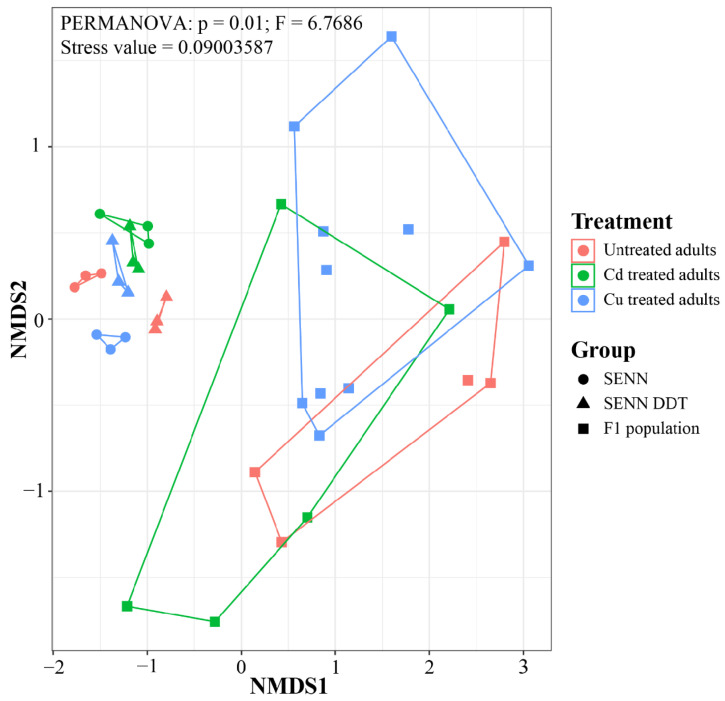
Beta diversity of laboratory and F_1_ population *An. arabiensis* adults exposed to metal pollutants as larvae. Diversity is presented as non-metric multidimensional scaling ordination, with comparisons between groups using a PERMANOVA analysis.

There was a significant difference in the Beta diversity of the laboratory strains and the F_1_ samples (PERMANOVA: *p* = 0.01, F = 6.77, Stress value = 0.08, R = 1.00). In the laboratory strains, the metal treatment resulted in Beta diversity that does not overlap. However, the F_1_ samples had a significantly greater Beta diversity than all the laboratory strains, with overlap between both treatments and the control (Figure 2). 

### 3.3. Overlapping Genera

When analysing the genera shared between the treated and untreated specimens within the same strain, SENN had 17 genera shared, SENN-DDT had 30, and F_1_ adults had 23. The treatment that resulted in the most unique strains differed between the strains. In SENN, larval copper treatment resulted in the most unique species, while the untreated had the least. In SENN-DDT, cadmium treatment resulted in the most unique genera while copper treatment resulted in the least unique species. The untreated and copper treatment differed by one unique genus. In the F_1_ adults, copper treatment resulted in the greatest number of unique genera (43 compared to 16 in untreated, and 5 for cadmium treatment). 

**Figure 3:**
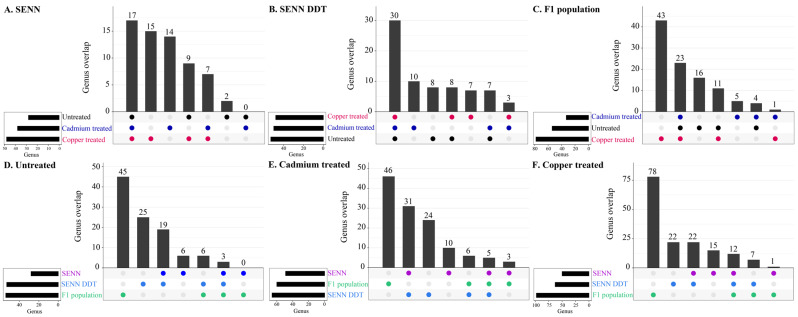
Overlapping genera of laboratory and wild adult An. arabiensis treated with metal as larvae. (**A**) Upset plot of overlapping genera in untreated *An. arabiensis*. (**B**) Upset plot of over-lapping genera in cadmium treated *An. arabiensis*. (**C**) Upset plot of overlapping genera in copper treated *An. arabiensis*. (**D**) Upset plot of overlapping genera in untreated *An. arabiensis*. (**E**) Upset plot of overlapping genera in cadmium-treated *An. arabiensis.* (**F**) Upset plot of overlapping genera in copper-treated *An. arabiensis.*

The overlapping genera were also examined in all three strains per metal treatment. In the untreated group, the F_1_ population had 45 unique genera compared to 25 in SENN-DDT and only 6 in SENN. There were only 3 shared genera between the three groups (*Allorhizobium-Neorhizobium-Pararhizobium-Rhizobium*, *Novosphingobium*, *Saccharimonadales*) (Figure 3A). In the cadmium-treated group, the F_1_ population had the most unique genera (46) compared to 24 in SENN-DDT and 10 in SENN. There were 5 genera shared between the groups after cadmium treatment (*Allorhizobium-Neorhizobium-Pararhizobium-Rhizobium*, *Novosphingobium*, *Pseudomonas*, *Rhodopseudomonas*, *Saccharimonadales*) (Figure 3B). Lastly, in the copper treatment cohorts, the F_1_ population had 78 unique genera compared to 22 in SENN-DDT and 15 in SENN. There were 12 genera shared between the three groups (*Comamonadaceae*, *Saccharimonadales*, *Sphingobacterium*, *Rahnella*, *Pseudarcicella*, *Novosphingobium*, *Elizabethkingia*, *Delftia*, *Bradyrhizobium*, *Bosea*, *Allorhizobium-Neorhizobium-Pararhizobium-Rhizobium*, *Acidovorax*) (Figure 3C). 

### 3.4. Differential Abundance

**Figure 4:**
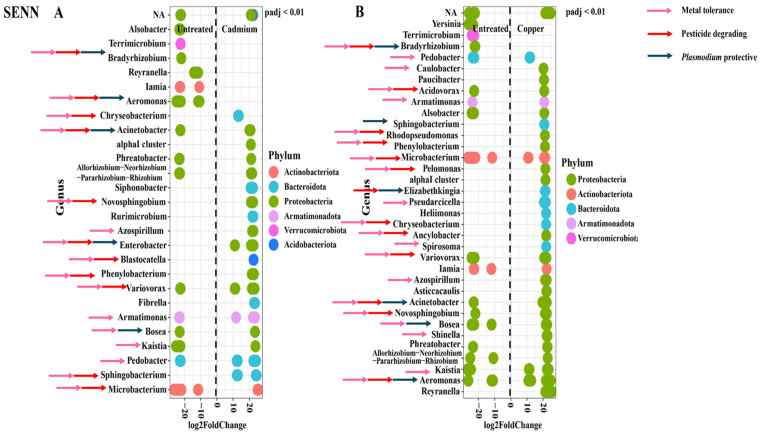
Comparison of the relative abundance of genera in the adult *An. arabiensis* SENN strain before and after metal treatment. (**A**) SENN untreated vs. SENN cadmium. (**B**) SENN untreated vs. SENN copper. Blue arrows indicate genera associated with *Plasmodium* protection. Red arrows indicate genera associated with insecticide resistance. Pink arrows indicate genera associated with metal tolerance.

In the SENN strain, cadmium treatment resulted in six unique genera being differentially abundant in the treatment cohorts. Five genera were differentially abundant in the untreated adults only. The remaining genera were abundant in both the treated and untreated groups. There were two *Plasmodium* protective genera unique to the untreated group, and one in the cadmium-treated group. Bacterial genera associated with insecticide resistance and metal tolerance were found in both treated and untreated groups. There were six genera associated with metal tolerance unique to the cadmium treatment compared to two in the untreated. For genera associated with insecticide resistance, there were five unique in the cadmium treatment compared to two in the untreated (Figure 4A). 

A similar pattern was observed with copper treatment. Although there were two *Plasmodium* protective genera unique to the treated group and only one in the untreated, insecticide resistance and metal tolerance-associated genera were found in both the treated and untreated groups. In the copper treatment, ten genera associated with metal tolerance were unique to the treatment group compared to one in the untreated group. For genera associated with insecticide resistance, there were six unique in the copper treatment compared to one in the untreated (Figure 4B).

**Figure 5:**
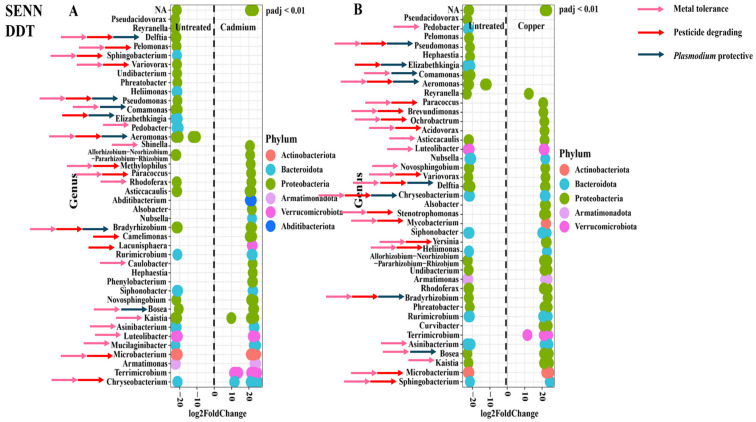
Comparison of the relative abundance of genera in the adult *An. arabiensis* SENN-DDT strain before and after metal treatment. (**A**) SENN-DDT untreated vs. SENN-DDT cadmium. (**B**) SENN-DDT untreated vs. SENN-DDT copper. Blue arrows indicate genera associated with *Plasmodium* protection. Red arrows indicate genera associated with insecticide resistance. Pink arrows indicate genera associated with metal tolerance.

The distribution pattern of the relatively abundant species was different in the SENN-DDT strain. There were twelve genera uniquely differentially abundant in the cadmium-treated group and fourteen in the untreated group. There were four unique *Plasmodium* protective genera in the untreated group but none in the treated group. There were eight metal protective genera unique to the untreated group, in contrast to four in the cadmium-treated group. Genera associated with insecticide resistance were uniquely differentially abundant in the untreated group with seven in comparison to two in the cadmium-treated group (Figure 5A). 

In the copper treatment, there were ten unique genera in the treated group compared to eight in the untreated group. Similar to the cadmium treatment, there were four *Plasmodium* protective genera unique to the untreated group while there were none in the cadmium-treated group. The patterns of unique genera were different for the copper treatment. While there were four unique metal protective genera unique to the untreated group, there were seven unique in the copper-treated group. Similarly, while there was a single genus associated with insecticide resistance unique to the untreated group, there were seven unique genera in the cadmium-treated group (Figure 5B). 

**Figure 6:**
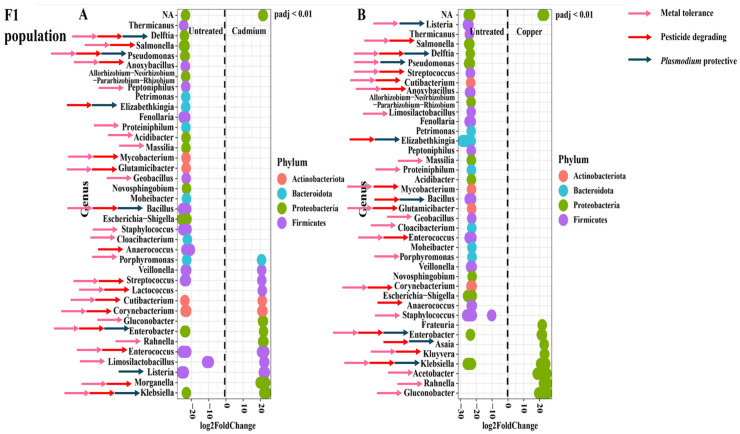
Comparison of the relative abundance of genera in the adult F_1_ *An. arabiensis* before and after metal treatment. (**A**) F_1_ untreated vs. F_1_ cadmium. (**B**) F_1_ untreated vs. F_1_ copper. Blue arrows indicate genera associated with *Plasmodium* protection. Red arrows indicate genera associated with insecticide resistance. Pink arrows indicate genera associated with metal tolerance.

The relative abundance profiles in the F_1_ adults were markedly different to those of laboratory strains. In the cadmium treatment, there were only two genera that were uniquely relatively abundant after treatment, compared to twenty-three unique genera in the untreated group. Therefore, most of the genera relatively abundant in the cadmium treatment were also relatively abundant in the untreated group. There were three *Plasmodium* protective genera unique to the untreated group but none in the cadmium-treated group. There were fourteen metal protective genera uniquely relatively abundant in the untreated group, but only three in the cadmium-treated group. Similarly, there were nine unique genera associated with insecticide resistance present in the untreated group but only two in the cadmium-treated group (Figure 6A).

These patterns were also present in the copper treatment. There were thirty-one genera uniquely relatively abundant in the untreated group but only five in the copper-treated group. There were five *Plasmodium* protective genera uniquely relatively abundant in the untreated group, but only one in the copper-treated group. In the untreated group, there were eighteen genera uniquely relatively abundant that were associated with metal protection, but only four in the copper-treated group. Finally, there were twelve genera associated with insecticide resistance uniquely relatively abundant in the untreated group but only a single genus uniquely relatively abundant in the copper-treated group (Figure 6B). 

## 4. Discussion

The larval environment is an important determinant of adult microbiota. However, despite increasing reports of adaptation of the *An. gambiae* complex adapting to breeding in polluted water, there is not a large body of data about the effect of these pollutants on the gut bacterial composition.

When comparing the overall alpha diversity of adults treated with metal as larvae, there was no significant difference from that of their untreated counterparts. However, within the metal treatments, there were small differences between the three groups. Cadmium treatment resulted in differences in species richness and diversity between the F_1_ population and SENN-DDT. This was similar to the copper treatment, although the Chao 1 richness index indicated a decrease in the richness of the F_1_ population compared to both strains unlike in the cadmium treatment. The lack of a marked difference in alpha diversity between treated and untreated samples is not unprecedented as it was also found in Pygmy grasshoppers that bred in mining areas [49]. Alpha diversity in copper-treated Chironomids did not differ from their untreated counterparts at lower concentrations but decreased at higher concentrations [50]. This suggests that the lack of differences seen in this experiment was potentially due to low-concentration exposure, as the concentrations used were at the acceptable concentration threshold for these metals [31].

Despite the lack of variation in alpha diversity, there was a marked difference in beta diversity after treatments. As expected, the F_1_ population had a greater beta diversity than that of the laboratory strains. While the metal-treated laboratory strains clustered together with their untreated counterparts, they did not overlap with the F_1_ population. The F_1_ population, by contrast, had a large overlap between the three groups. This suggests that while the F_1_ population had a greater diversity, the laboratory strains had a more distinctive bacterial diversity after treatment compared to the F_1_ population. There is not a large body of data to compare this to, but it is worth noting that copper exposure in Chironomids resulted in a similar beta diversity pattern [50].

A key aim of this study was to determine whether larval metal treatment of laboratory strains differing in insecticide-resistant phenotype and geographical origin to an F_1_ population from South Africa had similar bacterial dynamics in response to treatment. This would provide information on the utility of laboratory strains in understanding environmental effects on bacterial dynamics. This study indicated that there were large-scale differences between all three strains examined. For SENN, both metal treatments resulted in more unique genera represented (copper 15, cadmium 14, compared to 2 untreated). For SENN-DDT, the number of unique genera did not differ greatly (cadmium 10, untreated 8, copper 7). The F_1_ population, however, had 43 unique genera in response to copper treatment, which was markedly more than the 16 unique genera in the untreated group and only 5 in the cadmium-treated group. Despite these differences, the F_1_ population was more similar in response patterns to SENN than SENN-DDT. This suggests a role for the insecticide-resistant phenotype. The unselected SENN strain has low-level insecticide resistance and is more comparable in resistant phenotype to the F_1_ population [51] than SENN-DDT. This suggests that the selection for insecticide resistance may alter the dynamics of the bacterial response to polluted larval environments.

When comparing the genera between the three groups in response to treatment, the F_1_ population consistently had the greatest number of unique genera. By contrast, SENN always had the least number of unique genera. SENN and SENN-DDT always had more shared genera than either of the strains with the F_1_ population. There were very few genera shared between the three groups (3 in the untreated, 5 in the cadmium treatment and 12 in the copper treatment). This suggests a poorly conserved core microbiome shared between the three populations. This is consistent with previous studies that indicated clustering of mosquito microbiota by sampling location [52]. This is related to habitat-specific microbiota with geography driving biodiversity differences [53]. As such, the current study provides more information about patterns of responses rather than details about specific changes in the symbiont composition.

An examination of the differentially abundant genera shows a marked difference in response to metal treatment. In the SENN and SENN-DDT strains, the shared genera made up the greatest proportion of genera. This is reflected in the differential abundance, where the significantly abundant genera in the metal-treated individuals were similar in number to that of the untreated group. Furthermore, there were many shared genera differentially abundant in both treated and untreated groups. By contrast, there were many more differentially abundant genera in untreated F_1_ adults compared to the metal-treated groups. This is noteworthy due to the high numbers of unique genera in the F_1_ population, particularly in the copper-treated group. Yet, very few were differentially abundant. Copper is a bacterial micronutrient [54] and essential for insect physiology [55], and this could explain why copper treatment frequently resulted in the greatest number of unique genera and had fewer negative effects on the mosquito. This was true for SENN and the F_1_ population. For SENN-DDT, the number of unique genera did not differ greatly in any of the treatments. This again reinforces a link between the bacterial response and the insecticide-resistant phenotype. Crucially, the metal treatment, particularly in the F_1_ population, therefore increased the number of genera present, but this change in abundance was not significant. The effect of metal exposure on gut microbial composition has not been well examined in insects. Exposure to environmental metal decreases the diversity of gut microbiota vertebrates such as tree sparrows [56]. The diversity of gut microbiota was decreased in the Pygmy grasshopper *Eucriotettix oculatus* while increasing pathogenic bacteria [49]. Exposure to high metal concentrations reduced the abundance of dominant bacterial families in the black soldier fly *Hermetia illucens*. However, certain families were increased. These included *Brucellaceae*, *Enterobacteriaceae*, *Alcaligenaceae*, *Campylobacteraceae*, and *Enterococcaceae* [57]. Cadmium exposure decreased probiotic bacteria in the gut of the gypsy moth *Lymantria dispar*. This included *Weissella*, *Aeromonas*, and *Serratia*. Pathogenic bacteria, however, increased in abundance (*Stenotrophomonas*, *Gardnerella*, *Cutibacterium, Pluralibacter*, and *Tsukamurella*) [58]. None of the examples, however, are aquatic, like mosquito larvae. Chironomids are aquatic insects with a notable tolerance to pollutants. In the case of metal exposure here, specific genera were increased in response to specific metals. *Yersinia, Dysgonomonas, Delftia,* and *Enterococcus* were increased in response to chromium treatment. *Yersinia* and *Acinetobacter* were increased in response to copper treatment [50].

The most obvious suggestion is that the change in bacterial abundance was related to tolerating the metal pollutant. This was substantiated in the laboratory strains. The differentially abundant strains unique to the metal treatment tended to be more likely to be associated with metal tolerance. These included *Novosphingobium* [59], *Azospirillum* [60], *Blastocatella* [61], *Phenylobacterium* [62], and *Sphingobacterium* [63] in cadmium-treated SENN and *Pelomonas* [64], *Chryseobacterium* [65], *Ancylobacter* [66], *Spirosoma* [67], *Azospirilium* [68], *Rhodopseudomonas* [69], *Phenylobacterium* [62], and *Shinella* [70] in copper-treated SENN. In SENN-DDT cadmium-treated adults, the metal-tolerant genera *Shinella* [71], *Methylophilus* [72], *Paracoccus* [73], and *Caulobacter* [74] were uniquely relatively abundant. Similarly, *Paracoccus*, *Brevundimonas* [75], *Ochrobactrum* [76], *Acidovorax* [77], *Stenotrophomonas* [78], *Mycobacterium* [79], and *Yersinia* [80] were uniquely relatively abundant in copper-treated adults. Although genera associated with tolerance to metal were relatively abundant in both treated and untreated individuals, there were more metal-tolerant genera unique to the metal-treated individuals. This would suggest a role for these bacteria in surviving metal treatment. This, however, was not as clearly demonstrated in the F_1_ population where very few genera were uniquely relatively abundant. What was notable, however, was that despite the reduced number of relatively abundant genera, every single uniquely abundant genus in the metal treatment was associated with metal tolerance. For cadmium treatment, this was the genera *Lactococcus* [81], *Rahnella* [82], and *Morganella*. This is similar in copper-treated F_1_, where of seven uniquely relatively abundant genera in the metal-treated adults, four (*Kluyvera* [83], *Acetobacter* [84], *Rahnella* [82], and *Gluconobacter* [85]) were associated with metal tolerance.

What this suggests is that gut bacteria may be associated with the capacity to survive larval exposure to metal. This is more marked in the laboratory strains, but there is evidence that this happens in the F_1_ population as well. This is not unprecedented. There are variations in life history traits in laboratory strains and wild mosquitoes. This includes blood-feeding duration, oviposition behaviour, mating success, and swarming behaviour [86]. This is quite notable in the immune response. Insecticide-resistant *Culex pipiens* differed in phenoloxidase activity compared to susceptible counterparts. However, this was not present in wild populations [87]. Another study noted a decrease in immune function in insecticide-resistant *Cx. pipiens*, but again, this was not observed in their wild counterparts [88]. The association between larval exposure to metals and the relative abundance of metal-tolerant genera unique to metal treatment occurs in both laboratory strains and F_1_ populations. Even though the effect is more marked in the laboratory strains, it is present in the F_1_ adults as well. As such, it is likely that the effect occurs in the wild as well, although it is exaggerated in laboratory strains.

There is therefore a precedent for larval gut microbiota to protect from metal toxicants. However, the key question in this study relates to larval exposure to metals affecting mining for paratransgenesis candidates. In SENN, only one *Plasmodium* protective genus (*Enterobacter* [89]) was uniquely differentially abundant in the cadmium-treated individuals. Two were uniquely differentially abundant in untreated individuals compared to their cadmium-treated individuals (*Bradyrhizobium* [90], *Aeromonas* [91]), while the remaining protective genera were abundant in both untreated and cadmium-treated individuals (*Acinetobacter* [91], *Bosea* [90]). In copper-treated individuals, the *Plasmodium* protective genera *Sphingobacterium* [92] and *Elizabethkingia* [93] were more abundant than in their untreated counterparts. In the untreated group, *Bradyrhizobium* was the only protective genus uniquely differentially abundant compared to its copper-treated counterparts. *Aeromonas* and *Bosea* were abundant in both copper-treated and untreated SENN. This was in contrast to SENN-DDT where four genera were more abundant in untreated individuals compared to the cadmium-treated individuals (*Delftia* [94], *Pseudomonas* [95], *Comamonas* [96], and *Elizabethkingia*). There were no *Plasmodium* protective genera uniquely relatively abundant in the cadmium treatment. This was similar to the response after copper treatment where *Pseudomonas*, *Elizabethkingia*, *Comamonas*, and *Aeromonas* were uniquely relatively abundant in the untreated group and there were none in the copper-treated group. The response in the F_1_ group was similar to that of SENN-DDT. There were more *Plasmodium* protective genera in the untreated group uniquely relatively abundant compared to the cadmium-treated group (*Delftia*, *Pseudomonas*, *Elizabethkingia*, *Bacillus* [97]), while there were none uniquely relatively abundant in the cadmium-treated group. In the copper treatment, *Asaia* [98] was the only *Plasmodium* protective genus uniquely relatively abundant in the treated group. By contrast, *Listeria* [99], *Delftia*, *Pseudomonas*, *Elizabethkingia,* and *Bacillus* were protective genera uniquely relatively abundant in the untreated group. It is also worth noting, however, that *Gluconobacter*, a genus uniquely relatively abundant in both copper- and cadmium-treated F_1_ adults, is associated with increased activity of the *imd* immune pathway in *Drosophila* [100]. This immunological pathway is critical for protection against *Plasmodium* [101]. It is therefore worth noting that in the wild population, metal treatment reduced the amount of *Plasmodium* protective genera that were relatively abundant. This is something that needs to be considered when trying to mine native protective symbionts.

It has been observed that adapting to breeding in polluted environments has acted as a selection pressure for insecticide resistance [25,27]. This has also been demonstrated in SENN and SENN-DDT, where copper and cadmium exposure increased deltamethrin tolerance, even in the offspring of these adults that bred in clean water [34,35]. It would therefore be interesting to consider whether potentially pesticide-degrading bacteria play a role in this selection. Genera that have previously been associated with pesticide-degrading bacteria were found relatively abundant in both treated and untreated SENN for both metals. Only *Sphingobacterium* [102] was uniquely relatively abundant in cadmium-treated SENN. In the copper-treated SENN, *Elizabethkingia* [103], *Chryseobacterium* [104,105], *Rhodospseudomonas* [106], *Pelomonas* [107], *Phenylobacterium* [108], and *Ancylobacter* [109] were uniquely relatively abundant compared to only *Bradyrhizobium* [110] in the untreated group. The pesticide-degrading genera *Microbacterium* [111], *Variovorax* [112,113], *Acidovorax* [114], and *Aeromonas* [115] were found relatively abundant in both untreated and copper-treated samples. This was less marked in SENN-DDT, where only *Camelimonas* [116], *Paracoccus* [117], *Methylophilus* [111], and *Lacunisphaera* [118] were uniquely relatively abundant in the copper-treated individuals, while eight pesticide-degrading genera were unique to their untreated counterparts [104]. *Sphingobacterium* and *Microbacterium* were pesticide-degrading genera that were relatively abundant in copper-treated and untreated SENN-DDT. For copper-treated SENN-DDT, the degrading genera *Paracoccus* [117], *Brevundimonas* [119], *Ochrobactrum* [59], *Acidovorax* [120], *Mycobacterium* [121], *Stenotrophomonas* [122], and *Yersinia* [123] were uniquely relatively abundant in the treated group. Only two pesticide-degrading genera were uniquely relatively abundant in the untreated group compared to the copper-treated group, and five genera associated with resistance were abundant in both groups (*Sphingobacterium* [102], *Microbacterium*, *Bradyrhizobium*, and *Delftia* [124]). This suggests a pesticide-degrading bacteria may potentially be enriched by larval metal treatment, and this was particularly marked for copper treatment. This was not reflected in the F_1_ population, where although pesticide-degrading genera were present, they were often found in the untreated group or in both groups. For the cadmium-treated F_1_, only *Morganella* [125] and *Lactococcus* [126] were uniquely relatively abundant in the treated group. This was similar in the copper-treated group where only *Asaia* [127] and *Kluyvera* [120] were uniquely differentially abundant in the treated group. This suggests that in the wild population, metal treatment is not as strong a selection pressure for pesticide-degrading bacteria. This is another example of an exaggerated response in laboratory strains that are not replicated in the wild population. The role of metal selection on the abundance of pesticide-degrading bacteria in the F1 population would need to be confirmed by examining the change in abundance of subsequent generations.

This study does have a range of limitations. It could have been improved by comparing the F_1_ population to laboratory strains of closer geographical locations. Furthermore, to fully characterise the role of gut bacteria in the wild population, this would need to be followed by a few generations of breeding in polluted water. Secondly, to fully confirm the protective role of bacteria against larval metal exposure, bacteria in larvae need to be analysed. What has been observed in this study are the changes that occur in adults after larval exposure. It must also be noted that associations between the genera are the responses based on the literature. Therefore, although the relevant genera may previously have been associated with metal tolerance, insecticide resistance, and protection against *Plasmodium*, it is not guaranteed that this will be a direct translation. Crucially, it would be important to identify the relevant genera to species and strains. This would be essential for the establishment of paratransgenesis as an intervention strategy.

## 5. Conclusions

In conclusion, this study demonstrates limited utility for examining the effect of metal pollution on the microbial dynamics in *An. arabiensis*. Copper appears to result in a greater abundance of metal-tolerant genera. In general, there is evidence that gut bacteria could protect against larval metal intoxication, as the bacteria present in the adult represent a reduced diversity of what is present in the larvae. Furthermore, there is a precedent for this effect in other insects. Laboratory strains had an increased number of pesticide-degrading bacteria after metal treatment, but this was not represented in the F_1_ population. The number of *Plasmodium* protective genera in the F_1_ population after metal treatment was decreased. This suggests that mining for native paratransgenesis candidates where *An. arabiensis* breeds in polluted water may be less efficient than where the sampled mosquitoes breed in unpolluted water. This highlights the importance of considering the larval environment when searching for useful symbionts from local populations.

## Data Availability

The data presented in this study are available on request from the corresponding author.

## Supplementary Materials

The following supporting information can be downloaded at: https://www.mdpi.com/article/10.3390/tropicalmed9100249/s1. Table S1: ASVs identified in this study.
