# Supplementary material for: The Effect of Larval Exposure to Heavy Metals on the Gut Microbiota Composition of Adult Anopheles arabiensis (Diptera: Culicidae)
Source: Trop Med Infect Dis. 2024 Oct 21;9(10):249. doi: 10.3390/tropicalmed9100249 (PMC11510740; doi:10.3390/tropicalmed9100249)
Supplement: Supplementary file 1 [file tropicalmed-09-00249-s001.zip › tropicalmed-3205163-supplementary.pdf]

	Kingdom	Phylum	Class	Order	Family	Genus	Species
ASV1	Bacteria	Proteobacter	Gammaprote	Enterobacter	Yersiniaceae	Yersinia	
ASV2	Bacteria	Proteobacter	Gammaprote	Enterobacter	Yersiniaceae		
ASV3	Bacteria	Proteobacter	Gammaprote	Enterobacter	Yersiniaceae	Rahnella1	
ASV4	Bacteria	Proteobacter	Gammaprote	Enterobacter	Yersiniaceae	Yersinia	
ASV5	Bacteria	Proteobacter	Gammaprote	Enterobacter	Yersiniaceae	Rahnella1	
ASV6	Bacteria	Proteobacter	Gammaprote	Enterobacter	Yersiniaceae	Rahnella1	
ASV7	Bacteria	Proteobacter	Gammaprote	Pseudomona	Pseudomona	Pseudomona	veronii
ASV8	Bacteria	Proteobacter	Gammaprote	Pseudomona	Pseudomona	Pseudomona	veronii
ASV9	Bacteria	Proteobacter	Gammaprote	Pseudomona	Pseudomona	Pseudomona	veronii
ASV10	Bacteria	Proteobacter	Gammaprote	Enterobacter	Yersiniaceae		
ASV11	Bacteria	Proteobacter	Gammaprote	Enterobacter	Aeromonada	Aeromonas	
ASV12	Bacteria	Proteobacter	Gammaprote	Enterobacter	Yersiniaceae	Rahnella1	
ASV13	Bacteria	Proteobacter	Gammaprote	Enterobacter	Yersiniaceae	Yersinia	
ASV14	Bacteria	Proteobacter	Gammaprote	Enterobacter	Enterobacter	Enterobacter	
ASV15	Bacteria	Proteobacter	Gammaprote	Pseudomona	Pseudomona	Pseudomona	veronii
ASV16	Bacteria	Proteobacter	Alphaproteol	Reyranellales	Reyranellace	Reyranella	
ASV17	Bacteria	Proteobacter	Gammaprote	Enterobacter	Yersiniaceae	Yersinia	
ASV18	Bacteria	Proteobacter	Gammaprote	Enterobacter	Enterobacter	Enterobacter	
ASV19	Bacteria	Proteobacter	Gammaprote	Enterobacter	Yersiniaceae	Yersinia	
ASV20	Bacteria	Proteobacter	Gammaprote	Enterobacter	Yersiniaceae	Rahnella1	
ASV21	Bacteria	Proteobacter	Gammaprote	Enterobacter	Yersiniaceae		
ASV22	Bacteria	Proteobacter	Gammaprote	Enterobacter	Yersiniaceae	Yersinia	
ASV23	Bacteria	Proteobacter	Gammaprote	Enterobacter	Aeromonada	Aeromonas	
ASV24	Bacteria	Proteobacter	Gammaprote	Enterobacter	Yersiniaceae	Yersinia	
ASV25	Bacteria	Proteobacter	Gammaprote	Pseudomona	Pseudomona	Pseudomona	veronii
ASV26	Bacteria	Proteobacter	Gammaprote	Enterobacter	Yersiniaceae	Yersinia	aldovae
ASV27	Bacteria	Proteobacter	Alphaproteol	Reyranellales	Reyranellace	Reyranella	
ASV28	Bacteria	Proteobacter	Gammaprote	Enterobacter	Yersiniaceae	Rahnella1	
ASV29	Bacteria	Proteobacter	Gammaprote	Enterobacter	Yersiniaceae	Yersinia	
ASV30	Bacteria	Proteobacter	Gammaprote	Enterobacter	Enterobacter	Enterobacter	
ASV31	Bacteria	Proteobacter	Gammaprote	Enterobacter	Yersiniaceae		
ASV32	Bacteria	Proteobacter	Gammaprote	Pseudomona	Pseudomona	Pseudomona	veronii
ASV33	Bacteria	Proteobacter	Gammaprote	Enterobacter	Yersiniaceae	Yersinia	
ASV34	Bacteria	Proteobacter	Gammaprote	Enterobacter	Yersiniaceae		
ASV35	Bacteria	Bacteroidota	Bacteroidia	Flavobacteria	Weeksellace	Elizabethking	anophelis
ASV36	Bacteria	Bacteroidota	Bacteroidia	Flavobacteria	Weeksellace	Elizabethking	anophelis
ASV37	Bacteria	Bacteroidota	Bacteroidia	Flavobacteria	Weeksellace	Elizabethking	anophelis
ASV38	Bacteria	Proteobacter	Gammaprote	Enterobacter	Aeromonada	Aeromonas	encheleia
ASV39	Bacteria	Proteobacter	Gammaprote	Pseudomona	Pseudomona	Pseudomona	veronii
ASV40	Bacteria	Proteobacter	Gammaprote	Enterobacter	Yersiniaceae		
ASV41	Bacteria	Proteobacter	Gammaprote	Enterobacter	Yersiniaceae	Rahnella1	
ASV42	Bacteria	Proteobacter	Gammaprote	Enterobacter	Enterobacter	Klebsiella	
ASV43	Bacteria	Proteobacter	Gammaprote	Enterobacter	Yersiniaceae	Yersinia	
ASV44	Bacteria	Proteobacter	Gammaprote	Enterobacter	Yersiniaceae	Yersinia	aldovae
ASV45	Bacteria	Actinobacteri	Actinobacteri	Micrococcale	Microbacteri	Microbacteri	laevaniforma
ASV46	Bacteria	Proteobacter	Gammaprote	Enterobacter	Yersiniaceae	Rahnella1	
ASV47	Bacteria	Proteobacter	Gammaprote	Pseudomona	Pseudomona	Pseudomona	veronii
ASV48	Bacteria	Proteobacter	Alphaproteol	Reyranellales	Reyranellace	Reyranella	
ASV49	Bacteria	Proteobacter	Gammaprote	Enterobacter	Enterobacter	Klebsiella	

ASV50	Bacteria	Proteobacter	Gammaprote	Enterobacter	Yersiniaceae	Yersinia	
ASV51	Bacteria	Bacteroidota	Bacteroidia	Flavobacteria	Weeksellacea	Elizabethking	anophelis
ASV52	Bacteria	Armatimonac	Armatimonac	Armatimonac	Armatimonac	Armatimonas	
ASV53	Bacteria	Proteobacter	Gammaprote	Enterobacter	Enterobacter	Klebsiella	
ASV54	Bacteria	Proteobacter	Gammaprote	Enterobacter	Yersiniaceae	Yersinia	
ASV55	Bacteria	Proteobacter	Gammaprote	Pseudomona	Pseudomona	Pseudomona	veronii
ASV56	Bacteria	Proteobacter	Gammaprote	Enterobacter	Yersiniaceae	Rahnella1	
ASV57	Bacteria	Proteobacter	Gammaprote	Enterobacter	Yersiniaceae	Rahnella1	
ASV58	Bacteria	Proteobacter	Gammaprote	Enterobacter	Yersiniaceae	Yersinia	
ASV59	Bacteria	Proteobacter	Gammaprote	Enterobacter	Yersiniaceae	Rahnella1	
ASV60	Bacteria	Bacteroidota	Bacteroidia	Sphingobacte	Sphingobacte	Sphingobacte	multivorum
ASV61	Bacteria	Proteobacter	Gammaprote	Enterobacter	Yersiniaceae	Rahnella1	
ASV62	Bacteria	Bacteroidota	Bacteroidia	Sphingobacte	Sphingobacte	Sphingobacte	multivorum
ASV63	Bacteria	Proteobacter	Gammaprote	Enterobacter	Enterobacter	Enterobacter	
ASV64	Bacteria	Proteobacter	Gammaprote	Enterobacter	Yersiniaceae	Rahnella1	
ASV65	Bacteria	Proteobacter	Gammaprote	Enterobacter	Enterobacter	Enterobacter	roggenkampii
ASV66	Bacteria	Proteobacter	Gammaprote	Enterobacter	Yersiniaceae	Yersinia	
ASV67	Bacteria	Proteobacter	Gammaprote	Enterobacter	Yersiniaceae	Yersinia	
ASV68	Bacteria	Proteobacter	Gammaprote	Enterobacter	Enterobacter	Enterobacter	
ASV69	Bacteria	Bacteroidota	Bacteroidia	Flavobacteria	Weeksellacea	Chryseobacte	massiliae
ASV70	Eukaryota						
ASV71	Bacteria	Bacteroidota	Bacteroidia	Sphingobacte	Sphingobacte	Sphingobacte	multivorum
ASV72	Bacteria	Proteobacter	Gammaprote	Enterobacter	Enterobacter	Enterobacter	
ASV73	Bacteria	Proteobacter	Gammaprote	Enterobacter	Yersiniaceae	Yersinia	
ASV74	Bacteria	Proteobacter	Gammaprote	Pseudomona	Pseudomona	Pseudomonas	
ASV75	Bacteria	Proteobacter	Gammaprote	Enterobacter	Enterobacter	Klebsiella	quasipneumo
ASV76	Bacteria	Proteobacter	Gammaprote	Enterobacter	Aeromonada	Aeromonas	hydrophila
ASV77	Bacteria	Bacteroidota	Bacteroidia	Sphingobacte	Sphingobacte	Pedobacter	solisilvae
ASV78	Bacteria	Proteobacter	Gammaprote	Enterobacter	Enterobacter	Klebsiella	quasipneumo
ASV79	Bacteria	Proteobacter	Alphaproteol	Acetobactera	Acetobactera	Gluconobacte	frateurii
ASV80	Bacteria	Bacteroidota	Bacteroidia	Sphingobacte	Sphingobacte	Sphingobacte	multivorum
ASV81	Bacteria	Proteobacter	Gammaprote	Enterobacter	Yersiniaceae	Rahnella1	
ASV82	Bacteria	Bacteroidota	Bacteroidia	Flavobacteria	Weeksellacea	Elizabethking	anophelis
ASV83	Bacteria	Bacteroidota	Bacteroidia	Flavobacteria	Weeksellacea	Elizabethking	anophelis
ASV84	Bacteria	Proteobacter	Gammaprote	Enterobacter	Enterobacter	Enterobacter	
ASV85	Bacteria	Bacteroidota	Bacteroidia	Flavobacteria	Weeksellacea	Elizabethking	anophelis
ASV86	Bacteria	Proteobacter	Gammaprote	Enterobacter	Yersiniaceae	Rahnella1	
ASV87	Bacteria	Proteobacter	Gammaprote	Enterobacter	Enterobacter	Enterobacter	
ASV88	Bacteria	Proteobacter	Gammaprote	Enterobacter	Aeromonada	Aeromonas	encheleia
ASV89	Bacteria	Proteobacter	Gammaprote	Pseudomona	Pseudomona	Pseudomona	viridiflava
ASV90	Bacteria	Proteobacter	Alphaproteol	Reyranellales	Reyranellacea	Reyranella	
ASV91	Bacteria	Proteobacter	Gammaprote	Enterobacter	Yersiniaceae	Rahnella1	
ASV92	Bacteria	Proteobacter	Gammaprote	Enterobacter	Yersiniaceae	Rahnella1	
ASV93	Bacteria	Proteobacter	Alphaproteol	Sphingomona	Sphingomona	Sphingopyxis	terrae
ASV94	Bacteria	Bacteroidota	Bacteroidia	Sphingobacte	Sphingobacte	Sphingobacte	mizutaii
ASV95	Bacteria	Proteobacter	Gammaprote	Enterobacter	Enterobacter	Kluyvera	
ASV96	Bacteria	Bacteroidota	Bacteroidia	Sphingobacte	Sphingobacte	Sphingobacte	multivorum
ASV97	Bacteria	Proteobacter	Gammaprote	Enterobacter	Enterobacter	Klebsiella	quasipneumo
ASV98	Bacteria	Patescibacter	Saccharimona	Saccharimonadales			
ASV99	Bacteria	Actinobacteri	Actinobacteri	Micrococcale	Microbacteria	Microbacteriu	laevaniforma

ASV100	Bacteria	Proteobacter	Gammaprote	Enterobacter	Enterobacter	Kluyvera	
ASV101	Bacteria	Bacteroidota	Bacteroidia	Flavobacteria	Weeksellacea	Chryseobacterium	
ASV102	Bacteria	Proteobacter	Gammaprote	Enterobacter	Enterobacter	Klebsiella	michiganensis
ASV103	Bacteria	Bacteroidota	Bacteroidia	Sphingobacte	Sphingobacte	Sphingobacte	mizutaii
ASV104	Bacteria	Proteobacter	Gammaprote	Enterobacter	Aeromonada	Aeromonas	hydrophila
ASV105	Bacteria	Proteobacter	Alphaproteol	Acetobactera	Acetobactera	Acetobacter	pasteurianus
ASV106	Bacteria	Bacteroidota	Bacteroidia	Flavobacteria	Weeksellacea	Elizabethking	anophelis
ASV107	Bacteria	Bacteroidota	Bacteroidia	Flavobacteria	Weeksellacea	Elizabethking	anophelis
ASV108	Bacteria	Proteobacter	Gammaprote	Enterobacter	Enterobacter	Klebsiella	michiganensis
ASV109	Bacteria	Proteobacter	Gammaprote	Enterobacter	Yersiniaceae	Rahnella1	
ASV110	Bacteria	Proteobacter	Gammaprote	Enterobacter	Aeromonada	Aeromonas	encheleia
ASV111	Bacteria	Bacteroidota	Bacteroidia	Sphingobacte	Sphingobacte	Pedobacter	solisilvae
ASV112	Bacteria	Proteobacter	Alphaproteol	Sphingomona	Sphingomona	Sphingopyxis	terrae
ASV113	Bacteria	Proteobacter	Gammaprote	Enterobacter	Enterobacter	Kluyvera	
ASV114	Bacteria	Actinobacteri	Actinobacteri	Micrococcale	Microbacteria	Microbacteri	laevaniformai
ASV115	Bacteria	Proteobacter	Gammaprote	Enterobacter	Enterobacter	Klebsiella	michiganensis
ASV116	Bacteria	Proteobacter	Gammaprote	Enterobacter	Enterobacter	Klebsiella	michiganensis
ASV117	Bacteria	Proteobacter	Alphaproteol	Reyranellales	Reyranellacea	Reyranella	
ASV118	Bacteria	Firmicutes	Bacilli	Bacillales	Bacillaceae	Bacillus	
ASV119	Bacteria	Proteobacter	Gammaprote	Enterobacter	Enterobacter	Klebsiella	michiganensis
ASV120	Bacteria	Actinobacteri	Actinobacteri	Micrococcale	Microbacteria	Microbacteri	laevaniformai
ASV121	Bacteria	Proteobacter	Gammaprote	Enterobacter	Enterobacter	Cedecea	neteri
ASV122	Bacteria	Proteobacter	Gammaprote	Enterobacter	Enterobacter	Klebsiella	michiganensis
ASV123	Bacteria	Actinobacteri	Actinobacteri	Micrococcale	Microbacteria	Microbacteri	laevaniformai
ASV124	Bacteria	Proteobacter	Alphaproteol	Acetobactera	Acetobactera	Acetobacter	pasteurianus
ASV125	Bacteria	Proteobacter	Gammaprote	Enterobacter	Aeromonada	Aeromonas	
ASV126	Bacteria	Proteobacter	Gammaprote	Pseudomona	Pseudomona	Pseudomona	veronii
ASV127	Bacteria	Proteobacter	Gammaprote	Enterobacter	Yersiniaceae	Yersinia	
ASV128	Bacteria	Proteobacter	Alphaproteol	Rhizobiales	Rhizobiales Incertae Sedis		
ASV129	Bacteria	Patescibacter	Saccharimona	Saccharimonadales			
ASV130	Bacteria	Proteobacter	Alphaproteol	Acetobactera	Acetobactera	Gluconobacte	frateurii
ASV131	Bacteria	Bacteroidota	Bacteroidia	Flavobacteria	Weeksellacea	Elizabethking	anophelis
ASV132	Bacteria	Firmicutes	Bacilli	Bacillales	Bacillaceae	Bacillus	
ASV133	Bacteria	Bacteroidota	Bacteroidia	Flavobacteria	Weeksellacea	Elizabethking	anophelis
ASV134	Bacteria	Proteobacter	Gammaprote	Enterobacter	Enterobacter	Cedecea	neteri
ASV135	Bacteria	Bacteroidota	Bacteroidia	Sphingobacte	Sphingobacte	Sphingobacte	mizutaii
ASV136	Bacteria	Proteobacter	Alphaproteol	Reyranellales	Reyranellacea	Reyranella	
ASV137	Bacteria	Proteobacter	Alphaproteol	Reyranellales	Reyranellacea	Reyranella	
ASV138	Bacteria	Proteobacter	Gammaprote	Enterobacter	Enterobacter	Enterobacter	
ASV139	Bacteria	Firmicutes	Bacilli	Bacillales	Bacillaceae	Bacillus	intestinalis
ASV140	Bacteria	Proteobacter	Alphaproteol	Reyranellales	Reyranellacea	Reyranella	
ASV141	Bacteria	Proteobacter	Gammaprote	Enterobacter	Yersiniaceae	Yersinia	
ASV142	Bacteria	Proteobacter	Gammaprote	Enterobacter	Enterobacter	Klebsiella	quasipneumo
ASV143	Bacteria	Proteobacter	Gammaprote	Pseudomona	Pseudomona	Pseudomona	veronii
ASV144	Bacteria	Proteobacter	Alphaproteol	Acetobactera	Acetobactera	Asaia	krungthepens
ASV145	Bacteria	Proteobacter	Gammaprote	Enterobacter	Yersiniaceae	Yersinia	
ASV146	Bacteria	Proteobacter	Alphaproteol	Acetobactera	Acetobactera	Gluconobacte	frateurii
ASV147	Bacteria	Proteobacter	Alphaproteol	Rhizobiales	Kaistiaceae	Kaistia	
ASV148	Bacteria	Bacteroidota	Bacteroidia	Flavobacteria	Weeksellacea	Elizabethking	anophelis
ASV149	Bacteria	Actinobacteri	Actinobacteri	Micrococcale	Microbacteria	Microbacteri	laevaniformai

ASV150	Bacteria	Proteobacter	Gammaprote	Enterobacter	Enterobacteriaceae		
ASV151	Bacteria	Proteobacter	Alphaproteol	Acetobactera	Acetobactera	Acetobacter	pasteurianus
ASV152	Bacteria	Proteobacter	Gammaprote	Pseudomona	Pseudomona	Pseudomona	veronii
ASV153	Bacteria	Proteobacter	Alphaproteol	Sphingomona	Sphingomona	Sphingopyxis	terrae
ASV154	Bacteria	Proteobacter	Gammaprote	Enterobacter	Enterobacter	Enterobacter	
ASV155	Bacteria	Actinobacteri	Actinobacteri	Micrococcale	Microbacteri	Microbacteriu	laevaniforma
ASV156	Bacteria	Proteobacter	Gammaprote	Enterobacter	Aeromonada	Aeromonas	
ASV157	Bacteria	Proteobacter	Gammaprote	Enterobacter	Yersiniaceae	Yersinia	
ASV158	Bacteria	Proteobacter	Gammaprote	Enterobacter	Yersiniaceae	Yersinia	
ASV159	Bacteria	Proteobacter	Gammaprote	Enterobacter	Yersiniaceae	Rahnella1	
ASV160	Bacteria	Proteobacter	Gammaprote	Enterobacter	Enterobacter	Klebsiella	michiganensis
ASV161	Bacteria	Firmicutes	Bacilli	Lactobacillale	Enterococcac	Enterococcus	faecalis
ASV162	Bacteria	Firmicutes	Bacilli	Lactobacillale	Enterococcac	Enterococcus	faecalis
ASV163	Bacteria	Bacteroidota	Bacteroidia	Sphingobacte	Sphingobacte	Sphingobacte	mizutaii
ASV164	Bacteria	Firmicutes	Bacilli	Lactobacillale	Lactobacillac	Limosilactobacillus	
ASV165	Bacteria	Proteobacter	Gammaprote	Pseudomona	Pseudomona	Pseudomona	veronii
ASV166	Bacteria	Proteobacter	Alphaproteol	Rhizobiales	Rhizobiales Incertae Sedis		
ASV167	Bacteria	Bacteroidota	Bacteroidia	Flavobacteria	Weeksellacea	Elizabethking	anophelis
ASV168	Bacteria	Proteobacter	Alphaproteol	Acetobactera	Acetobactera	Acetobacter	pasteurianus
ASV169	Bacteria	Proteobacter	Alphaproteol	Acetobactera	Acetobactera	Acetobacter	pasteurianus
ASV170	Bacteria	Proteobacter	Alphaproteol	Acetobactera	Acetobactera	Gluconobacte	frateurii
ASV171	Bacteria	Bacteroidota	Bacteroidia	Sphingobacte	Sphingobacte	Pedobacter	solisilvae
ASV172	Bacteria	Proteobacter	Gammaprote	Enterobacter	Yersiniaceae	Yersinia	aldovae
ASV173	Bacteria	Firmicutes	Bacilli	Lactobacillale	Enterococcac	Enterococcus	faecalis
ASV174	Bacteria	Proteobacter	Alphaproteol	Acetobactera	Acetobactera	Gluconobacte	frateurii
ASV175	Bacteria	Proteobacter	Alphaproteol	Reyranellales	Reyranellacea	Reyranella	
ASV176	Bacteria	Proteobacter	Gammaprote	Enterobacter	Enterobacter	Enterobacter	
ASV177	Bacteria	Firmicutes	Bacilli	Lactobacillale	Lactobacillac	Limosilactobacillus	
ASV178	Bacteria	Proteobacter	Gammaprote	Enterobacter	Yersiniaceae	Rahnella1	
ASV179	Bacteria	Proteobacter	Alphaproteol	Reyranellales	Reyranellacea	Reyranella	
ASV180	Bacteria	Proteobacter	Gammaprote	Enterobacter	Enterobacteriaceae		
ASV181	Bacteria	Proteobacter	Gammaprote	Enterobacter	Enterobacter	Klebsiella	michiganensis
ASV182	Bacteria	Proteobacter	Gammaprote	Pseudomona	Pseudomona	Pseudomona	veronii
ASV183	Bacteria	Proteobacter	Gammaprote	Enterobacter	Yersiniaceae	Yersinia	
ASV184	Bacteria	Proteobacter	Alphaproteol	Rhizobiales	Rhizobiaceae	Allorhizobium-Neorhizobiu	
ASV185	Bacteria	Proteobacter	Gammaprote	Enterobacter	Enterobacter	Klebsiella	
ASV186	Bacteria	Firmicutes	Bacilli	Lactobacillale	Lactobacillac	Limosilactobacillus	
ASV187	Bacteria	Firmicutes	Bacilli	Bacillales	Bacillaceae	Bacillus	intestinalis
ASV188	Bacteria	Proteobacter	Alphaproteol	Rhizobiales	Kaistiaceae	Kaistia	
ASV189	Bacteria	Proteobacter	Gammaprote	Enterobacter	Enterobacter	Klebsiella	quasipneumo
ASV190	Bacteria	Proteobacter	Gammaprote	Enterobacter	Yersiniaceae	Rahnella1	
ASV191	Bacteria	Proteobacter	Gammaprote	Pseudomona	Pseudomona	Pseudomona	veronii
ASV192	Bacteria	Proteobacter	Alphaproteol	Rhizobiales	Kaistiaceae	Kaistia	
ASV193	Bacteria	Proteobacter	Alphaproteol	Rhizobiales	Rhizobiales Incertae Sedis		
ASV194	Bacteria	Proteobacter	Gammaprote	Enterobacter	Enterobacter	Klebsiella	variicola
ASV195	Bacteria	Actinobacteri	Actinobacteri	Micrococcale	Microbacteri	Microbacteriu	laevaniforma
ASV196	Bacteria	Bacteroidota	Bacteroidia	Flavobacteria	Weeksellacea	Elizabethking	anophelis
ASV197	Bacteria	Proteobacter	Gammaprote	Enterobacter	Enterobacter	Enterobacter	
ASV198	Bacteria	Proteobacter	Gammaprote	Enterobacter	Morganellace	Morganella	morganii
ASV199	Bacteria	Proteobacter	Gammaprote	Enterobacter	Yersiniaceae	Yersinia	aldovae

ASV200	Bacteria	Proteobacter	Gammaprote	Pseudomona	Pseudomona	Pseudomona	veronii
ASV201	Bacteria	Proteobacter	Gammaprote	Enterobacter	Enterobacter	Klebsiella	
ASV202	Bacteria	Proteobacter	Gammaprote	Enterobacter	Enterobacter	Klebsiella	variicola
ASV203	Bacteria	Firmicutes	Bacilli	Lactobacillale	Listeriaceae	Listeria	
ASV204	Bacteria	Proteobacter	Alphaproteol	Rhizobiales	Kaistiaceae	Kaistia	
ASV205	Bacteria	Proteobacter	Gammaprote	Enterobacter	Enterobacteriaceae		
ASV206	Bacteria	Proteobacter	Alphaproteol	Acetobactera	Acetobactera	Gluconobacte	frateurii
ASV207	Bacteria	Proteobacter	Gammaprote	Enterobacter	Morganellace	Morganella	morganii
ASV208	Bacteria	Firmicutes	Bacilli	Lactobacillale	Lactobacillac	Limosilactobacillus	
ASV209	Bacteria	Actinobacteri	Actinobacteri	Micrococcale	Microbacteri	Microbacterium	
ASV210	Bacteria	Actinobacteri	Actinobacteri	Micrococcale	Microbacteri	Microbacterium	
ASV211	Bacteria	Proteobacter	Gammaprote	Enterobacter	Yersiniaceae	Yersinia	
ASV212	Bacteria	Proteobacter	Gammaprote	Enterobacter	Aeromonada	Aeromonas	
ASV213	Bacteria	Actinobacteri	Actinobacteri	Micrococcale	Microbacteri	Microbacterium	
ASV214	Bacteria	Firmicutes	Bacilli	Staphylococc	Staphylococc	Staphylococcus	
ASV215	Bacteria	Proteobacter	Alphaproteol	Acetobactera	Acetobactera	Asaia	krungthepens
ASV216	Bacteria	Proteobacter	Alphaproteol	Rhizobiales	Rhizobiaceae		
ASV217	Bacteria	Proteobacter	Gammaprote	Enterobacter	Enterobacter	Cedecea	neteri
ASV218	Bacteria	Proteobacter	Alphaproteol	Rhizobiales	Kaistiaceae	Kaistia	
ASV219	Eukaryota						
ASV220	Bacteria	Proteobacter	Alphaproteol	Rhizobiales	Kaistiaceae	Kaistia	
ASV221	Bacteria	Proteobacter	Alphaproteol	Acetobactera	Acetobactera	Acetobacter	pasteurianus
ASV222	Bacteria	Bacteroidota	Bacteroidia	Sphingobacte	Sphingobacte	Pedobacter	
ASV223	Bacteria	Proteobacter	Gammaprote	Enterobacter	Morganellace	Morganella	morganii
ASV224	Bacteria	Proteobacter	Alphaproteol	Rhizobiales	Rhizobiaceae	Allorhizobium-Neorhizobiu	
ASV225	Bacteria	Proteobacter	Alphaproteol	Acetobactera	Acetobactera	Gluconobacte	frateurii
ASV226	Bacteria	Proteobacter	Gammaprote	Enterobacter	Morganellace	Morganella	morganii
ASV227	Bacteria	Proteobacter	Alphaproteol	Rhizobiales	Kaistiaceae	Kaistia	
ASV228	Bacteria	Proteobacter	Alphaproteol	Acetobactera	Acetobactera	Asaia	krungthepens
ASV229	Bacteria	Proteobacter	Alphaproteol	Rhizobiales	Rhizobiales Incertae Sedis		
ASV230	Bacteria	Proteobacter	Alphaproteol	Rhizobiales	Beijerinckiace	Bosea	
ASV231	Bacteria	Proteobacter	Alphaproteol	Rhizobiales	Kaistiaceae	Kaistia	
ASV232	Bacteria	Proteobacter	Alphaproteol	Acetobactera	Acetobactera	Gluconobacte	frateurii
ASV233	Bacteria	Proteobacter	Gammaprote	Pseudomona	Pseudomona	Pseudomonas	
ASV234	Bacteria	Actinobacteri	Actinobacteri	Micrococcale	Microbacteri	Microbacteri	laevaniforma
ASV235	Bacteria	Proteobacter	Gammaprote	Enterobacter	Enterobacter	Klebsiella	quasipneumo
ASV236	Bacteria	Actinobacteri	Actinobacteri	Micrococcale	Microbacteri	Microbacteri	laevaniforma
ASV237	Bacteria	Proteobacter	Gammaprote	Enterobacter	Enterobacter	Klebsiella	
ASV238	Bacteria	Proteobacter	Gammaprote	Enterobacter	Enterobacter	Klebsiella	michiganensis
ASV239	Bacteria	Proteobacter	Gammaprote	Enterobacter	Enterobacter	Klebsiella	variicola
ASV240	Bacteria	Proteobacter	Gammaprote	Enterobacter	Morganellace	Morganella	morganii
ASV241	Bacteria	Firmicutes	Bacilli	Lactobacillale	Listeriaceae	Listeria	
ASV242	Bacteria	Proteobacter	Gammaprote	Enterobacter	Yersiniaceae	Yersinia	
ASV243	Bacteria	Bacteroidota	Bacteroidia	Sphingobacte	Sphingobacte	Sphingobacte	multivorum
ASV244	Bacteria	Proteobacter	Alphaproteol	Rhizobiales	Kaistiaceae	Kaistia	
ASV245	Bacteria	Firmicutes	Bacilli	Staphylococc	Staphylococc	Staphylococcus	
ASV246	Bacteria	Firmicutes	Bacilli	Lactobacillale	Enterococcac	Enterococcus	faecalis
ASV247	Bacteria	Proteobacter	Gammaprote	Enterobacter	Enterobacter	Klebsiella	michiganensis
ASV248	Bacteria	Firmicutes	Bacilli	Lactobacillale	Listeriaceae	Listeria	
ASV249	Bacteria	Proteobacter	Gammaprote	Enterobacter	Enterobacter	Escherichia-Shigella	

ASV250	Bacteria	Proteobacter	Gammaprote	Enterobacter	Morganellace	Morganella	morganii
ASV251	Bacteria	Proteobacter	Gammaprote	Enterobacter	Enterobacter	Klebsiella	
ASV252	Bacteria	Proteobacter	Gammaprote	Enterobacter	Enterobacter	Escherichia-Shigella	
ASV253	Bacteria	Proteobacter	Gammaprote	Enterobacter	Enterobacteriaceae		
ASV254	Bacteria	Bacteroidota	Bacteroidia	Sphingobacte	Sphingobacte	Sphingobacte	mizutaii
ASV255	Bacteria	Firmicutes	Bacilli	Lactobacillale	Listeriaceae	Listeria	
ASV256	Bacteria	Actinobacteri	Actinobacteri	Micrococcale	Microbacteri	Microbacterium	
ASV257	Bacteria	Proteobacter	Gammaprote	Burkholderial	Comamonad	Variovorax	paradoxus
ASV258	Bacteria	Proteobacter	Gammaprote	Enterobacter	Enterobacter	Klebsiella	quasipneumo
ASV259	Bacteria	Proteobacter	Alphaproteol	Rhizobiales	Beijerinckiace	Bosea	
ASV260	Bacteria	Proteobacter	Gammaprote	Enterobacter	Enterobacter	Cedecea	neteri
ASV261	Bacteria	Proteobacter	Alphaproteol	Rhizobiales	Kaistiaceae	Kaistia	
ASV262	Bacteria	Proteobacter	Alphaproteol	Rhizobiales	Rhizobiaceae		
ASV263	Bacteria	Proteobacter	Alphaproteol	Acetobactera	Acetobactera	Gluconobacte	frateurii
ASV264	Bacteria	Proteobacter	Gammaprote	Enterobacter	Aeromonada	Aeromonas	
ASV265	Bacteria	Proteobacter	Alphaproteol	Acetobactera	Acetobactera	Gluconobacte	frateurii
ASV266	Bacteria	Proteobacter	Gammaprote	Enterobacter	Enterobacter	Klebsiella	variicola
ASV267	Bacteria	Proteobacter	Alphaproteol	Rhizobiales	Kaistiaceae	Kaistia	
ASV268	Bacteria	Proteobacter	Gammaprote	Enterobacter	Enterobacter	Klebsiella	
ASV269	Bacteria	Firmicutes	Bacilli	Staphylococc	Staphylococc	Staphylococcus	
ASV270	Bacteria	Proteobacter	Alphaproteol	Rhizobiales	Rhizobiaceae		
ASV271	Bacteria	Proteobacter	Gammaprote	Enterobacter	Enterobacter	Klebsiella	
ASV272	Bacteria	Proteobacter	Alphaproteol	Acetobactera	Acetobactera	Gluconacetol	liquefaciens
ASV273	Bacteria	Actinobacteri	Actinobacteri	Micrococcale	Microbacteri	Microbacteri	maritypicum
ASV274	Bacteria	Actinobacteri	Actinobacteri	Micrococcale	Microbacteri	Microbacteri	laevaniforma
ASV275	Bacteria	Firmicutes	Bacilli	Staphylococc	Staphylococc	Staphylococcus	
ASV276	Bacteria	Proteobacter	Gammaprote	Enterobacter	Enterobacter	Escherichia-S	fergusonii
ASV277	Bacteria	Proteobacter	Gammaprote	Enterobacter	Enterobacteriaceae		
ASV278	Bacteria	Proteobacter	Gammaprote	Pseudomona	Pseudomona	Pseudomona	psychrophila
ASV279	Bacteria	Firmicutes	Bacilli	Lactobacillale	Listeriaceae	Listeria	
ASV280	Bacteria	Proteobacter	Gammaprote	Enterobacter	Enterobacteriaceae		
ASV281	Bacteria	Proteobacter	Alphaproteol	Acetobactera	Acetobactera	Acetobacter	pasteurianus
ASV282	Bacteria						
ASV283	Bacteria	Firmicutes	Bacilli	Staphylococc	Staphylococc	Staphylococcus	
ASV284	Bacteria	Proteobacter	Alphaproteol	Acetobactera	Acetobactera	Acetobacter	pasteurianus
ASV285	Bacteria	Proteobacter	Gammaprote	Enterobacter	Enterobacter	Klebsiella	quasipneumo
ASV286	Bacteria	Proteobacter	Gammaprote	Enterobacter	Enterobacter	Klebsiella	quasipneumo
ASV287	Bacteria	Actinobacteri	Actinobacteri	Micrococcale	Microbacteri	Microbacteri	laevaniforma
ASV288	Bacteria	Proteobacter	Gammaprote	Pseudomona	Pseudomona	Pseudomona	viridiflava
ASV289	Bacteria	Proteobacter	Alphaproteol	Rhizobiales	Kaistiaceae	Kaistia	
ASV290	Bacteria	Bacteroidota	Bacteroidia	Flavobacteria	Weeksellacea	Elizabethking	anophelis
ASV291	Bacteria	Proteobacter	Gammaprote	Enterobacter	Yersiniaceae	Rahnella1	
ASV292	Bacteria	Proteobacter	Gammaprote	Pseudomona	Pseudomona	Pseudomona	psychrophila
ASV293	Bacteria	Proteobacter	Gammaprote	Pseudomona	Pseudomona	Pseudomona	viridiflava
ASV294	Bacteria	Proteobacter	Gammaprote	Enterobacter	Enterobacter	Klebsiella	michiganensi
ASV295	Bacteria	Proteobacter	Gammaprote	Enterobacter	Enterobacter	Klebsiella	quasipneumo
ASV296	Bacteria	Proteobacter	Gammaprote	Enterobacter	Enterobacteriaceae		
ASV297	Bacteria	Bacteroidota	Bacteroidia	Sphingobacte	Sphingobacte	Sphingobacte	mizutaii
ASV298	Bacteria	Firmicutes	Bacilli	Bacillales	Bacillaceae	Bacillus	intestinalis
ASV299	Bacteria	Actinobacteri	Actinobacteri	Micrococcale	Microbacteri	Microbacteri	laevaniforma

ASV300	Bacteria	Firmicutes	Bacilli	Staphylococc	Staphylococc	Staphylococcus	
ASV301	Bacteria	Proteobacter	Gammaprote	Enterobacter	Enterobacter	Escherichia-Shigella	
ASV302	Bacteria	Proteobacter	Gammaprote	Pseudomona	Pseudomona	Pseudomona	psychrophila
ASV303	Bacteria	Firmicutes	Bacilli	Lactobacillale	Listeriaceae	Listeria	
ASV304	Bacteria	Actinobacteri	Actinobacteri	Micrococcale	Microbacteria	Microbacterium	
ASV305	Bacteria	Proteobacter	Gammaprote	Enterobacter	Aeromonada	Aeromonas	
ASV306	Bacteria	Proteobacter	Alphaproteol	Acetobactera	Acetobactera	Gluconobacte	frateurii
ASV307	Bacteria	Firmicutes	Bacilli	Lactobacillale	Listeriaceae	Listeria	
ASV308	Bacteria	Proteobacter	Gammaprote	Pseudomona	Pseudomona	Pseudomona	psychrophila
ASV309	Bacteria	Proteobacter	Gammaprote	Enterobacter	Enterobacter	Klebsiella	oxytoca
ASV310	Bacteria	Proteobacter	Alphaproteol	Rhizobiales	Rhizobiales Ir	Phreatobacter	
ASV311	Bacteria	Bacteroidota	Bacteroidia	Flavobacteria	Flavobacteria	Flavobacteriu	lindanitolerar
ASV312	Bacteria	Bacteroidota	Bacteroidia	Sphingobacte	Sphingobacte	Sphingobacte	multivorum
ASV313	Bacteria	Firmicutes	Bacilli	Staphylococc	Staphylococc	Staphylococcus	
ASV314	Bacteria	Proteobacter	Gammaprote	Burkholderial	Comamonada	Comamonas	testosteroni
ASV315	Bacteria	Proteobacter	Alphaproteol	Rhizobiales	Beijerinckiace	Bosea	
ASV316	Bacteria	Proteobacter	Gammaprote	Pseudomona	Pseudomona	Pseudomona	lutea
ASV317	Bacteria	Proteobacter	Alphaproteol	Acetobactera	Acetobactera	Gluconobacte	frateurii
ASV318	Bacteria	Proteobacter	Alphaproteol	Rhizobiales	Kaistiaceae	Kaistia	
ASV319	Bacteria	Firmicutes	Bacilli	Lactobacillale	Lactobacillace	Limosilactobacillus	
ASV320	Bacteria	Proteobacter	Alphaproteol	Rhizobiales	Rhizobiales Ir	Phreatobacter	
ASV321	Bacteria	Proteobacter	Gammaprote	Enterobacter	Morganellace	Morganella	morganii
ASV322	Bacteria	Proteobacter	Gammaprote	Burkholderial	Comamonada	Comamonas	testosteroni
ASV323	Bacteria	Actinobacteri	Actinobacteri	Micrococcale	Microbacteria	Microbacterium	
ASV324	Bacteria	Proteobacter	Alphaproteol	Rhizobiales	Rhizobiales Ir	Phreatobacter	
ASV325	Bacteria	Proteobacter	Gammaprote	Enterobacter	Enterobacter	Klebsiella	quasipneumo
ASV326	Bacteria	Firmicutes	Bacilli	Staphylococc	Staphylococc	Staphylococc	aureus
ASV327	Bacteria	Firmicutes	Bacilli	Bacillales	Bacillaceae	Bacillus	
ASV328	Bacteria	Proteobacter	Gammaprote	Enterobacter	Enterobacter	Escherichia-Shigella	
ASV329	Bacteria	Firmicutes	Bacilli	Lactobacillale	Listeriaceae	Listeria	
ASV330	Bacteria	Bacteroidota	Bacteroidia	Sphingobacte	Sphingobacte	Sphingobacte	multivorum
ASV331	Bacteria	Proteobacter	Gammaprote	Enterobacter	Enterobacter	Escherichia-Shigella	
ASV332	Bacteria	Proteobacter	Alphaproteol	Rhizobiales	Xanthobacter	Ancylobacter	
ASV333	Bacteria	Proteobacter	Gammaprote	Enterobacter	Enterobacter	Enterobacter	
ASV334	Bacteria	Actinobacteri	Actinobacteri	Micrococcale	Microbacteria	Microbacteriu	laevaniformar
ASV335	Bacteria	Bacteroidota	Bacteroidia	Cytophagales			
ASV336	Bacteria	Proteobacter	Gammaprote	Pseudomona	Moraxellacea	Acinetobacte	lwoffii
ASV337	Bacteria	Proteobacter	Alphaproteol	Rhizobiales	Beijerinckiace	Bosea	
ASV338	Bacteria	Bacteroidota	Bacteroidia	Cytophagales	Spirosomacea	Fibrella	aestuarina
ASV339	Bacteria	Proteobacter	Alphaproteol	Sphingomona	Sphingomona	Sphingomonas	
ASV340	Bacteria	Bacteroidota	Bacteroidia	Cytophagales	Spirosomacea	Fibrella	aestuarina
ASV341	Bacteria	Proteobacter	Gammaprote	Pseudomona	Pseudomona	Pseudomona	aeruginosa
ASV342	Bacteria	Proteobacter	Gammaprote	Enterobacter	Enterobacter	Salmonella	
ASV343	Bacteria	Proteobacter	Gammaprote	Enterobacter	Enterobacteriaceae		
ASV344	Bacteria	Bacteroidota	Bacteroidia	Sphingobacte	Sphingobacte	Sphingobacte	multivorum
ASV345	Bacteria	Bacteroidota	Bacteroidia	Flavobacteria	Weeksellacea	Chryseobacterium	
ASV346	Bacteria	Bacteroidota	Bacteroidia	Sphingobacte	Sphingobacte	Pedobacter	solisilvae
ASV347	Bacteria	Bacteroidota	Bacteroidia	Sphingobacte	Sphingobacte	Sphingobacte	multivorum
ASV348	Bacteria	Bacteroidota	Bacteroidia	Chitinophaga	Chitinophaga	Rurimicrobium	
ASV349	Bacteria	Firmicutes	Bacilli	Lactobacillale	Streptococca	Streptococcu	thermophilus

ASV350	Bacteria	Firmicutes	Bacilli	Bacillales	Bacillaceae	Bacillus	
ASV351	Bacteria	Bacteroidota	Bacteroidia	Cytophagales	Spirosomace	Fibrella	aestuarina
ASV352	Bacteria	Proteobacter	Gammaprote	Pseudomona	Pseudomona	Pseudomona	aeruginosa
ASV353	Bacteria	Proteobacter	Gammaprote	Enterobacter	Enterobacter	Klebsiella	aerogenes
ASV354	Bacteria	Proteobacter	Gammaprote	Burkholderial	Comamonad	Variovorax	paradoxus
ASV355	Bacteria	Proteobacter	Gammaprote	Pseudomona	Pseudomona	Pseudomona	aeruginosa
ASV356	Bacteria	Proteobacter	Gammaprote	Enterobacter	Enterobacter	Salmonella	
ASV357	Bacteria						
ASV358	Bacteria	Bacteroidota	Bacteroidia	Cytophagales			
ASV359	Bacteria	Proteobacter	Gammaprote	Enterobacter	Enterobacter	Klebsiella	oxytoca
ASV360	Bacteria	Proteobacter	Gammaprote	Burkholderial	Comamonad	Variovorax	paradoxus
ASV361	Bacteria	Bacteroidota	Bacteroidia	Cytophagales	Spirosomace	Fibrella	aestuarina
ASV362	Bacteria	Proteobacter	Gammaprote	Pseudomona	Moraxellacea	Acinetobacte	lwoffii
ASV363	Bacteria	Proteobacter	Alphaproteol	Rhizobiales	Rhizobiales Ir	Phreatobacter	
ASV364	Bacteria	Proteobacter	Gammaprote	Enterobacter	Enterobacter	Escherichia-Shigella	
ASV365	Bacteria	Firmicutes	Bacilli	Staphylococc	Staphylococc	Staphylococc	aureus
ASV366	Bacteria	Proteobacter	Alphaproteol	Acetobactera	Acetobactera	Acetobacter	pasteurianus
ASV367	Bacteria	Proteobacter	Gammaprote	Enterobacter	Enterobacter	Klebsiella	
ASV368	Bacteria	Proteobacter	Gammaprote	Enterobacter	Enterobacter	Salmonella	
ASV369	Bacteria	Firmicutes	Bacilli	Lactobacillale	Lactobacillac	Limosilactobacillus	
ASV370	Bacteria	Firmicutes	Bacilli	Bacillales	Bacillaceae	Bacillus	intestinalis
ASV371	Bacteria	Proteobacter	Gammaprote	Pseudomona	Moraxellacea	Acinetobacter	
ASV372	Bacteria	Firmicutes	Bacilli	Lactobacillale	Streptococca	Streptococcu	thermophilus
ASV373	Bacteria	Proteobacter	Gammaprote	Xanthomona	Rhodanobact	Frateuria	aurantia
ASV374	Bacteria	Bacteroidota	Bacteroidia	Cytophagales			
ASV375	Bacteria	Actinobacteri	Actinobacteri	Corynebacter	Corynebacter	Corynebacter	amycolatum
ASV376	Bacteria	Firmicutes	Bacilli	Lactobacillale	Streptococca	Streptococcu	thermophilus
ASV377	Bacteria	Firmicutes	Bacilli	Lactobacillale	Lactobacillac	Limosilactobacillus	
ASV378	Bacteria	Proteobacter	Alphaproteol	Sphingomona	Sphingomona	Novosphingobium	
ASV379	Bacteria	Proteobacter	Gammaprote	Enterobacter	Enterobacter	Klebsiella	
ASV380	Bacteria	Actinobacteri	Acidimicrobii	Microtrichale	Iamiaceae	Iamia	
ASV381	Bacteria	Proteobacter	Gammaprote	Enterobacter	Enterobacter	Klebsiella	quasipneumo
ASV382	Bacteria	Proteobacter	Gammaprote	Enterobacter	Enterobacter	Escherichia-Shigella	
ASV383	Bacteria	Proteobacter	Alphaproteol	Rhizobiales	Xanthobacter	Ancylobacter	
ASV384	Bacteria	Proteobacter	Gammaprote	Enterobacter	Enterobacter	Klebsiella	
ASV385	Bacteria	Bacteroidota	Bacteroidia	Flavobacteria	Blattabacteri	Blattabacterium	
ASV386	Bacteria	Firmicutes	Negativicutes	Veillonellales	Veillonellace	Veillonella	atypica
ASV387	Bacteria	Actinobacteri	Actinobacteri	Micrococcale	Microbacteri	Leucobacter	
ASV388	Bacteria	Proteobacter	Gammaprote	Enterobacter	Enterobacter	Salmonella	
ASV389	Bacteria	Proteobacter	Gammaprote	Enterobacter	Enterobacter	Enterobacter	
ASV390	Bacteria	Bacteroidota	Bacteroidia	Sphingobacte	Sphingobacte	Sphingobacte	multivorum
ASV391	Bacteria	Proteobacter	Gammaprote	Enterobacter	Enterobacter	Enterobacter	
ASV392	Eukaryota						
ASV393	Bacteria	Firmicutes	Bacilli	Staphylococc	Staphylococc	Staphylococcus	
ASV394	Bacteria	Proteobacter	Gammaprote	Enterobacter	Enterobacter	Klebsiella	
ASV395	Bacteria	Proteobacter	Alphaproteol	Rhizobiales	Kaistiaceae	Kaistia	
ASV396	Bacteria	Firmicutes	Bacilli	Lactobacillale	Lactobacillac	Limosilactobacillus	
ASV397	Bacteria	Bacteroidota	Bacteroidia	Cytophagales			
ASV398	Bacteria	Proteobacter	Gammaprote	Pseudomona	Moraxellacea	Acinetobacte	lwoffii
ASV399	Bacteria	Bacteroidota	Bacteroidia	Cytophagales	Spirosomace	Fibrella	aestuarina

ASV400	Bacteria						
ASV401	Bacteria	Actinobacteri	Actinobacteri	Micrococcale	Microbacteri	Microbacterium	
ASV402	Bacteria	Proteobacter	Gammaprote	Enterobacter	Enterobacter	Klebsiella	michiganensis
ASV403	Bacteria	Proteobacter	Gammaprote	Pseudomona	Moraxellacea	Acinetobacter	
ASV404	Bacteria	Bacteroidota	Bacteroidia	Bacteroidetes	VC2.1 Bac22		
ASV405	Bacteria	Proteobacter	Gammaprote	Pseudomona	Moraxellacea	Acinetobacte	soli
ASV406	Bacteria	Proteobacter	Gammaprote	Burkholderial	Comamonada	Comamonas	testosteroni
ASV407	Bacteria	Proteobacter	Gammaprote	Enterobacter	Enterobacter	Enterobacter	
ASV408	Bacteria	Firmicutes	Bacilli	Staphylococc	Staphylococc	Staphylococcus	
ASV409	Bacteria	Proteobacter	Alphaproteol	Rhizobiales	Rhizobiaceae	Shinella	
ASV410	Bacteria	Proteobacter	Gammaprote	Enterobacter	Enterobacter	Enterobacter	soli
ASV411	Bacteria	Proteobacter	Gammaprote	Pseudomona	Pseudomona	Pseudomona	aeruginosa
ASV412	Bacteria	Proteobacter	Gammaprote	Pseudomona	Moraxellacea	Acinetobacte	lwoffii
ASV413	Bacteria	Proteobacter	Alphaproteol	Sphingomona	Sphingomona	Novosphingobium	
ASV414	Bacteria	Proteobacter	Gammaprote	Enterobacter	Aeromonada	Aeromonas	encheleia
ASV415	Bacteria	Firmicutes	Bacilli	Staphylococc	Staphylococc	Staphylococcus	
ASV416	Bacteria	Actinobacteri	Acidimicrobii	Microtrichale	Iamiaceae	Iamia	
ASV417	Bacteria	Proteobacter	Alphaproteol	Caulobactera	Caulobactera	Asticcacaulis	excentricus
ASV418	Bacteria	Proteobacter	Gammaprote	Pseudomona	Pseudomona	Pseudomona	aeruginosa
ASV419	Bacteria	Bacteroidota	Bacteroidia	Flavobacteria	Weeksellacea	Elizabethking	anophelis
ASV420	Bacteria	Proteobacter	Gammaprote	Pseudomona	Moraxellacea	Acinetobacte	soli
ASV421	Bacteria	Bacteroidota	Bacteroidia	Flavobacteria	Blattabacteri	Blattabacterium	
ASV422	Bacteria	Proteobacter	Gammaprote	Pseudomona	Pseudomona	Pseudomona	aeruginosa
ASV423	Bacteria	Proteobacter	Alphaproteol	Azospirillales	Azospirillacea	Azospirillum	lipoferum
ASV424	Bacteria	Bacteroidota	Bacteroidia	Flavobacteria	Blattabacteri	Blattabacterium	
ASV425	Bacteria	Actinobacteri	Acidimicrobii	Microtrichale	Iamiaceae	Iamia	
ASV426	Bacteria	Bacteroidota	Bacteroidia	Cytophagales			
ASV427	Bacteria	Firmicutes	Bacilli	Bacillales	Bacillaceae	Anoxybacillus	flavithermus
ASV428	Bacteria	Proteobacter	Gammaprote	Enterobacter	Enterobacter	Klebsiella	michiganensis
ASV429	Bacteria	Proteobacter	Alphaproteol	Rhizobiales	Rhizobiaceae	Allorhizobium-Neorhizobiu	
ASV430	Bacteria	Proteobacter	Alphaproteol	Rhizobiales	Rhizobiaceae	Allorhizobium-Neorhizobiu	
ASV431	Bacteria	Proteobacter	Alphaproteol	Rhizobiales	Rhizobiaceae	Shinella	
ASV432	Bacteria	Proteobacter	Gammaprote	Burkholderial	Alcaligenacea	Achromobact	piechaudii
ASV433	Bacteria	Proteobacter	Alphaproteol	Rhizobiales	Beijerinckiace	Bosea	
ASV434	Bacteria	Proteobacter	Gammaprote	Enterobacter	Enterobacter	Klebsiella	quasipneumo
ASV435	Bacteria	Proteobacter	Alphaproteol	Caulobactera	Caulobactera	Phenylobacterium	
ASV436	Bacteria	Proteobacter	Alphaproteol	Acetobactera	Acetobactera	Asaia	krungthepens
ASV437	Bacteria	Proteobacter	Gammaprote	Burkholderial	Comamonada	Variovorax	paradoxus
ASV438	Bacteria	Proteobacter	Gammaprote	Enterobacter	Enterobacter	Klebsiella	
ASV439	Bacteria	Proteobacter	Gammaprote	Pseudomona	Pseudomona	Pseudomona	aeruginosa
ASV440	Eukaryota						
ASV441	Bacteria	Proteobacter	Gammaprote	Enterobacter	Enterobacter	Enterobacter	kobei
ASV442	Bacteria	Bacteroidota	Bacteroidia	Cytophagales	Spirosomacea	Fibrella	aestuarina
ASV443	Bacteria	Firmicutes	Bacilli	Bacillales	Bacillaceae	Bacillus	intestinalis
ASV444	Bacteria	Proteobacter	Gammaprote	Pseudomona	Moraxellacea	Enhydrobacter	
ASV445	Bacteria	Firmicutes	Negativicutes	Veillonellales	Veillonellacea	Veillonella	
ASV446	Bacteria	Proteobacter	Gammaprote	Enterobacter	Enterobacter	Klebsiella	
ASV447	Bacteria	Proteobacter	Gammaprote	Enterobacter	Enterobacter	Klebsiella	
ASV448	Bacteria	Proteobacter	Alphaproteol	Sphingomona	Sphingomona	Novosphingobium	
ASV449	Bacteria	Proteobacter	Gammaprote	Enterobacter	Enterobacter	Klebsiella	

ASV450	Bacteria	Proteobacter	Gammaprote	Pseudomona	Moraxellacea	Acinetobacte	soli
ASV451	Bacteria	Armatimonac	Armatimonac	Armatimonac	Armatimonac	Armatimonas	
ASV452	Bacteria	Proteobacter	Gammaprote	Enterobacter	Enterobacter	Enterobacter	
ASV453	Bacteria	Proteobacter	Alphaproteol	Rhizobiales	Rhizobiales Ir	Phreatobacter	
ASV454	Bacteria	Proteobacter	Alphaproteol	Caulobactera	Caulobactera	Phenylobacterium	
ASV455	Bacteria	Firmicutes	Bacilli	Staphylococc	Staphylococc	Staphylococcus	
ASV456	Bacteria	Proteobacter	Gammaprote	Enterobacterales			
ASV457	Bacteria	Proteobacter	Gammaprote	Enterobacter	Aeromonada	Aeromonas	
ASV458	Bacteria						
ASV459	Bacteria	Proteobacter	Gammaprote	Enterobacter	Enterobacteriaceae		
ASV460	Bacteria	Proteobacter	Alphaproteol	Rhizobiales	Rhizobiaceae	Allorhizobium	rhizogenes
ASV461	Bacteria	Proteobacter	Gammaprote	Enterobacter	Enterobacter	Klebsiella	
ASV462	Bacteria	Proteobacter	Gammaprote	Enterobacter	Enterobacteriaceae		
ASV463	Bacteria	Bacteroidota	Bacteroidia	Cytophagales			
ASV464	Bacteria	Proteobacter	Gammaprote	Burkholderial	Comamonad	Variovorax	paradoxus
ASV465	Bacteria	Firmicutes	Bacilli	Lactobacillale	Lactobacillac	Limosilactobacillus	
ASV466	Bacteria	Proteobacter	Alphaproteol	Rhizobiales	Rhizobiaceae	Allorhizobium-Neorhizobiu	
ASV467	Bacteria	Bacteroidota	Bacteroidia	Cytophagales			
ASV468	Bacteria	Bacteroidota	Bacteroidia	Chitinophaga	Chitinophaga	Taibaiella	
ASV469	Bacteria	Proteobacter	Gammaprote	Enterobacter	Enterobacter	Klebsiella	quasipneumo
ASV470	Bacteria	Proteobacter	Gammaprote	Enterobacter	Enterobacter	Klebsiella	quasipneumo
ASV471	Bacteria	Proteobacter	Gammaprote	Pseudomona	Pseudomona	Pseudomona	aeruginosa
ASV472	Bacteria	Firmicutes	Bacilli	Lactobacillale	Listeriaceae	Listeria	
ASV473	Bacteria	Proteobacter	Alphaproteol	Caulobactera	Caulobactera	Phenylobacterium	
ASV474	Bacteria	Proteobacter	Alphaproteol	Rhizobiales	Rhizobiales Incertae Sedis		
ASV475	Bacteria	Proteobacter	Gammaprote	Enterobacter	Enterobacter	Klebsiella	oxytoca
ASV476	Bacteria	Proteobacter	Alphaproteol	Acetobactera	Acetobactera	Gluconacetol	liquefaciens
ASV477	Bacteria	Proteobacter	Gammaprote	Enterobacter	Enterobacteriaceae		
ASV478	Bacteria	Proteobacter	Alphaproteol	Rhizobiales	Rhizobiales Ir	Phreatobacter	
ASV479	Bacteria	Proteobacter	Alphaproteol	Rhizobiales	Rhizobiaceae	Mesorhizobiu	thiogangeticu
ASV480	Bacteria	Proteobacter	Gammaprote	Enterobacter	Enterobacteriaceae		
ASV481	Bacteria	Proteobacter	Alphaproteol	Rhizobiales	Rhizobiaceae	Allorhizobium-Neorhizobiu	
ASV482	Bacteria	Proteobacter	Gammaprote	Enterobacter	Enterobacteriaceae		
ASV483	Bacteria	Firmicutes	Bacilli	Lactobacillale	Enterococcac	Enterococcus	
ASV484	Bacteria	Bacteroidota	Bacteroidia	Cytophagales	Spirosomace	Spirosoma	aerolatum
ASV485	Bacteria	Armatimonac	Armatimonac	Armatimonadales			
ASV486	Bacteria	Bacteroidota	Bacteroidia	Cytophagales	Spirosomace	Pseudarcicell	hirudinis
ASV487	Bacteria	Proteobacter	Alphaproteol	Rhizobiales	Beijerinckiace	Bosea	
ASV488	Bacteria	Proteobacter	Gammaprote	Enterobacter	Enterobacter	Salmonella	
ASV489	Bacteria	Firmicutes	Bacilli	Staphylococc	Staphylococc	Staphylococcus	
ASV490	Bacteria	Firmicutes	Bacilli	Bacillales	Bacillaceae	Bacillus	intestinalis
ASV491	Bacteria	Proteobacter	Gammaprote	Enterobacter	Enterobacter	Klebsiella	quasipneumo
ASV492	Bacteria	Proteobacter	Gammaprote	Enterobacter	Enterobacteriaceae		
ASV493	Bacteria	Proteobacter	Gammaprote	Enterobacter	Enterobacteriaceae		
ASV494	Bacteria	Proteobacter	Gammaprote	Enterobacter	Enterobacter	Klebsiella	
ASV495	Bacteria	Proteobacter	Gammaprote	Enterobacter	Enterobacter	Enterobacter	
ASV496	Bacteria	Proteobacter	Gammaprote	Enterobacter	Morganellace	Morganella	morganii
ASV497	Bacteria						
ASV498	Bacteria	Bacteroidota	Bacteroidia	Flavobacteria	Weeksellacea	Chryseobacte	indologenes
ASV499	Bacteria	Proteobacter	Gammaprote	Enterobacter	Morganellace	Morganella	morganii

ASV500	Bacteria	Actinobacteri	Actinobacteri	Corynebacter	Corynebacter	Corynebacter	ureicelerivora
ASV501	Bacteria	Proteobacter	Gammaprote	Enterobacter	Enterobacteriaceae		
ASV502	Bacteria	Actinobacteri	Actinobacteri	Micrococcale	Microbacteri	Microbacterium	
ASV503	Bacteria	Proteobacter	Gammaprote	Enterobacter	Enterobacter	Enterobacter	kobei
ASV504	Bacteria	Firmicutes	Clostridia	Peptostrepto	Family XI	Ezakiella	
ASV505	Bacteria	Proteobacter	Alphaproteol	Rhizobiales	Xanthobacteraceae		
ASV506	Bacteria	Proteobacter	Gammaprote	Enterobacter	Enterobacter	Klebsiella	
ASV507	Bacteria	Proteobacter	Gammaprote	Enterobacter	Enterobacter	Kluyvera	
ASV508	Bacteria	Proteobacter	Gammaprote	Enterobacter	Enterobacter	Salmonella	virus
ASV509	Bacteria						
ASV510	Bacteria	Proteobacter	Gammaprote	Burkholderial	Oxalobactera	Duganella	
ASV511	Bacteria	Acidobacteric	Blastocatellia	Blastocatellal	Blastocatella	Blastocatella	
ASV512	Bacteria	Firmicutes	Bacilli	Lactobacillale	Listeriaceae	Listeria	
ASV513	Bacteria	Firmicutes	Clostridia	Peptostrepto	Family XI	Fenollaria	
ASV514	Bacteria	Proteobacter	Alphaproteol	Rhizobiales	Rhizobiales Ir	Phreatobacter	
ASV515	Bacteria	Proteobacter	Gammaprote	Enterobacter	Enterobacter	Salmonella	enterica
ASV516	Bacteria	Firmicutes	Clostridia	Peptostrepto	Family XI	Ezakiella	
ASV517	Bacteria	Proteobacter	Alphaproteol	Sphingomona	Sphingomona	Novosphingobium	
ASV518	Bacteria	Firmicutes	Bacilli	Lactobacillale	Streptococca	Streptococcus	
ASV519	Bacteria	Proteobacter	Alphaproteol	Rhizobiales	Devosiaceae	Devosia	
ASV520	Bacteria	Firmicutes	Bacilli	Lactobacillale	Streptococca	Streptococcus	
ASV521	Bacteria	Proteobacter	Gammaprote	Pseudomona	Moraxellacea	Acinetobacte	soli
ASV522	Bacteria	Bacteroidota	Bacteroidia	Flavobacteria	Weeksellacea	Elizabethking	anophelis
ASV523	Bacteria	Firmicutes	Clostridia	Peptostrepto	Family XI	Fenollaria	
ASV524	Bacteria	Actinobacteri	Actinobacteri	Corynebacter	Corynebacter	Corynebacterium	
ASV525	Bacteria	Proteobacter	Alphaproteol	Caulobactera	Caulobactera	Phenylobacterium	
ASV526	Bacteria	Proteobacter	Alphaproteol	Acetobactera	Acetobactera	Asaia	krungthepens
ASV527	Bacteria	Firmicutes	Bacilli	Bacillales	Bacillaceae	Bacillus	intestinalis
ASV528	Bacteria	Bacteroidota	Bacteroidia	Flavobacteria	Weeksellacea	Elizabethking	anophelis
ASV529	Bacteria	Proteobacter	Gammaprote	Burkholderial	Oxalobactera	Duganella	
ASV530	Bacteria	Bacteroidota	Bacteroidia	Chitinophaga	Chitinophaga	Heliimonas	saccharivorar
ASV531	Bacteria	Firmicutes	Bacilli	Lactobacillale	Enterococcac	Enterococcus	faecalis
ASV532	Bacteria	Bacteroidota	Bacteroidia	Chitinophaga	Chitinophaga	Taibaiella	
ASV533	Bacteria	Proteobacter	Alphaproteol	Rhizobiales	Beijerinckiace	Bosea	
ASV534	Bacteria	Proteobacter	Gammaprote	Burkholderial	Oxalobactera	Herbaspirillur	huttiense
ASV535	Bacteria	Firmicutes	Bacilli	Staphylococc	Staphylococc	Staphylococcus	
ASV536	Bacteria	Proteobacter	Alphaproteol	Rhizobiales	Beijerinckiace	Bosea	
ASV537	Eukaryota						
ASV538	Bacteria	Actinobacteri	Actinobacteri	Corynebacter	Corynebacter	Corynebacter	coyleae
ASV539	Bacteria						
ASV540	Bacteria	Proteobacter	Gammaprote	Enterobacter	Enterobacter	Klebsiella	quasipneumo
ASV541	Bacteria	Actinobacteri	Acidimicrobii	Microtrichale	Iamiaceae	Iamia	
ASV542	Bacteria	Proteobacter	Gammaprote	Enterobacter	Morganellace	Morganella	morganii
ASV543	Bacteria	Proteobacter	Gammaprote	Pseudomona	Moraxellacea	Acinetobacte	soli
ASV544	Bacteria	Firmicutes	Bacilli	Thermicanale	Thermicanac	Thermicanus	
ASV545	Bacteria						
ASV546	Bacteria	Firmicutes	Bacilli	Lactobacillale	Enterococcac	Enterococcus	faecalis
ASV547							
ASV548	Bacteria	Proteobacter	Alphaproteol	Azospirillales	Azospirillacea	Azospirillum	
ASV549	Bacteria	Proteobacter	Alphaproteol	Sphingomona	Sphingomona	Novosphingobium	

ASV550	Bacteria	Proteobacter	Gammaprote	Enterobacter	Enterobacteriaceae		
ASV551	Bacteria	Acidobacteric	Blastocatellia	Blastocatellal	Blastocatellaceae		
ASV552	Bacteria	Bacteroidota	Bacteroidia	Chitinophaga	Chitinophaga	Rurimicrobium	
ASV553	Bacteria	Proteobacter	Gammaprote	Burkholderial	Comamonada	Variovorax	
ASV554	Bacteria	Proteobacter	Gammaprote	Pseudomona	Pseudomona	Pseudomona	lutea
ASV555	Bacteria	Proteobacter	Alphaproteol	Rhodobactera	Rhodobactera	Paracoccus	
ASV556	Bacteria	Proteobacter	Alphaproteol	Rhizobiales	Rhizobiaceae	Allorhizobium	daejeonense
ASV557	Bacteria	Proteobacter	Gammaprote	Enterobacter	Enterobacter	Klebsiella	oxytoca
ASV558	Bacteria	Firmicutes	Bacilli	Lactobacillale	Enterococcac	Enterococcus	faecalis
ASV559	Bacteria	Proteobacter	Gammaprote	Enterobacter	Enterobacter	Klebsiella	quasipneumo
ASV560	Bacteria	Proteobacter	Gammaprote	Enterobacter	Enterobacteriaceae		
ASV561	Bacteria						
ASV562	Bacteria	Proteobacter	Gammaprote	Enterobacter	Aeromonada	Aeromonas	encheleia
ASV563	Bacteria	Proteobacter	Gammaprote	Enterobacter	Enterobacter	Salmonella	
ASV564	Bacteria	Proteobacter	Gammaprote	Enterobacter	Enterobacter	Klebsiella	
ASV565	Bacteria	Proteobacter	Alphaproteol	Rhizobiales	Rhizobiales Ir	Alsobacter	
ASV566	Bacteria	Proteobacter	Alphaproteol	Rhizobiales	Rhizobiaceae	Shinella	
ASV567	Bacteria	Proteobacter	Gammaprote	Enterobacter	Enterobacteriaceae		
ASV568	Bacteria	Proteobacter	Gammaprote	Enterobacter	Enterobacter	Salmonella	
ASV569	Bacteria	Firmicutes	Clostridia	Peptostrepto	Family XI	Anaerococcus	
ASV570	Bacteria	Proteobacter	Gammaprote	Enterobacter	Enterobacteriaceae		
ASV571	Bacteria	Proteobacter	Gammaprote	Enterobacter	Enterobacteriaceae		
ASV572	Bacteria	Proteobacter	Alphaproteol	Acetobactera	Acetobactera	Asaia	krungthepens
ASV573	Bacteria	Proteobacter	Gammaprote	Enterobacter	Enterobacter	Klebsiella	oxytoca
ASV574	Bacteria	Proteobacter	Alphaproteol	Rhizobiales	Xanthobacter	Bradyrhizobiu	elkanii
ASV575	Bacteria	Firmicutes	Bacilli	Lactobacillale	Enterococcac	Enterococcus	faecalis
ASV576	Bacteria	Firmicutes	Bacilli	Lactobacillale	Listeriaceae	Listeria	
ASV577	Eukaryota						
ASV578	Bacteria	Firmicutes	Bacilli	Lactobacillale	Enterococcac	Enterococcus	faecalis
ASV579	Bacteria	Proteobacter	Gammaprote	Burkholderial	Oxalobactera	Herbaspirillur	huttiense
ASV580	Bacteria	Proteobacter	Gammaprote	Enterobacter	Enterobacteriaceae		
ASV581	Bacteria	Firmicutes	Bacilli	Lactobacillale	Enterococcac	Enterococcus	
ASV582	Bacteria	Bacteroidota	Bacteroidia	Chitinophaga	Chitinophaga	Rurimicrobium	
ASV583	Bacteria	Proteobacter	Alphaproteol	Rhizobiales	Rhizobiales Ir	Alsobacter	
ASV584	Bacteria	Proteobacter	Alphaproteol	Caulobactera	Caulobactera	Phenylobacterium	
ASV585	Bacteria	Bacteroidota	Bacteroidia	Cytophagales	Spirosomacea	Spirosoma	aerolatum
ASV586	Bacteria	Proteobacter	Gammaprote	Enterobacter	Morganellace	Morganella	morganii
ASV587	Bacteria	Actinobacteri	Actinobacteri	Micrococcale	Microbacteria	Microbacterium	
ASV588	Bacteria	Proteobacter	Alphaproteol	Rhizobiales			
ASV589	Bacteria	Bacteroidota	Bacteroidia	Flavobacteria	Blattabacteria	Blattabacterium	
ASV590	Bacteria	Proteobacter	Gammaprote	Burkholderial	Comamonada	Paucibacter	
ASV591	Bacteria	Proteobacter	Alphaproteol	Rhizobiales	Rhizobiaceae	Allorhizobium	rhizogenes
ASV592	Bacteria	Bacteroidota	Bacteroidia	Flavobacteria	Flavobacteria	Flavobacteriu	lindanitolerar
ASV593	Bacteria	Proteobacter	Alphaproteol	Caulobactera	Caulobactera	Asticcacaulis	excentricus
ASV594	Bacteria	Firmicutes	Clostridia	Peptostrepto	Family XI	Fenollaria	
ASV595	Bacteria	Bacteroidota	Bacteroidia	Bacteroidales	Porphyromor	Porphyromor	bennonis
ASV596	Bacteria	Proteobacter	Alphaproteol	Azospirillales	Azospirillacea	Azospirillum	
ASV597	Bacteria	Proteobacter	Alphaproteol	Rhizobiales	Rhizobiaceae	Allorhizobium-Neorhizobiur	
ASV598	Bacteria	Proteobacter	Gammaprote	Burkholderial	Comamonada	Tepidimonas	fonticaldi
ASV599	Bacteria	Proteobacteria					

ASV600	Bacteria	Proteobacter	Gammaprote	Enterobacter	Enterobacter	Salmonella	
ASV601	Bacteria	Proteobacter	Gammaprote	Pseudomona	Moraxellacea	Acinetobacte	baylyi
ASV602	Bacteria	Bacteroidota	Bacteroidia	Bacteroidetes	VC2.1 Bac22		
ASV603	Bacteria	Proteobacter	Gammaprote	Enterobacter	Enterobacter	Kluyvera	
ASV604	Bacteria	Proteobacter	Gammaprote	Burkholderial	Comamonadaceae		
ASV605	Bacteria						
ASV606	Bacteria	Bacteroidota	Bacteroidia	Cytophagales	Cytophagacea	Siphonobacte	aquaeclarae
ASV607	Bacteria	Firmicutes	Clostridia	Peptostrepto	Family XI	Finegoldia	magna
ASV608	Bacteria	Proteobacter	Gammaprote	Enterobacter	Enterobacter	Klebsiella	
ASV609	Bacteria	Actinobacteri	Acidimicrobii	Microtrichale	Iamiaceae	Iamia	
ASV610	Bacteria	Proteobacter	Gammaprote	Burkholderial	Oxalobactera	Undibacteriu	squillarum
ASV611	Bacteria	Armatimonac	Armatimonac	Armatimonadales			
ASV612	Bacteria	Proteobacter	Alphaproteol	Rhizobiales	Xanthobacteraceae		
ASV613	Bacteria	Proteobacter	Gammaprote	Enterobacter	Enterobacter	Salmonella	
ASV614	Bacteria	Proteobacter	Gammaprote	Burkholderial	Oxalobactera	Herbaspirillu	huttiense
ASV615	Bacteria	Bacteroidota	Bacteroidia	Cytophagales	Spirosomacea	Spirosoma	aerolatum
ASV616	Bacteria	Firmicutes	Bacilli	Lactobacillale	Enterococcac	Enterococcus	faecalis
ASV617	Bacteria	Firmicutes	Bacilli	Lactobacillale	Enterococcac	Enterococcus	
ASV618	Eukaryota						
ASV619	Bacteria	Bacteroidota	Bacteroidia	Chitinophaga	Chitinophaga	Rurimicrobium	
ASV620	Bacteria	Proteobacter	Gammaprote	Enterobacter	Enterobacter	Enterobacter	
ASV621	Bacteria	Verrucomicro	Verrucomicro	Chthoniobact	Terrimicrobia	Terrimicrobium	
ASV622	Bacteria	Firmicutes	Bacilli	Lactobacillale	Enterococcac	Enterococcus	faecalis
ASV623	Bacteria	Firmicutes	Bacilli	Staphylococc	Staphylococc	Staphylococcus	
ASV624	Bacteria	Proteobacter	Gammaprote	Enterobacter	Enterobacter	Klebsiella	
ASV625	Bacteria	Proteobacter	Alphaproteol	Rhizobiales	Beijerinckiace	Methylobacterium-Methylo	
ASV626	Bacteria	Actinobacteri	Actinobacteri	Micrococcale	Microbacteria	Microbacterium	
ASV627	Bacteria	Proteobacter	Gammaprote	Pseudomona	Moraxellacea	Acinetobacte	baylyi
ASV628	Bacteria	Proteobacter	Alphaproteol	Rhizobiales	Rhizobiales Incertae Sedis		
ASV629	Bacteria	Proteobacter	Gammaprote	Enterobacter	Enterobacteriaceae		
ASV630	Bacteria						
ASV631	Bacteria	Proteobacter	Alphaproteol	Rhizobiales	Rhizobiaceae		
ASV632	Bacteria	Proteobacter	Alphaproteol	Caulobactera	Caulobactera	Caulobacter	vibrioides
ASV633	Bacteria	Proteobacter	Alphaproteol	Rhizobiales	Rhizobiales Ir	Phreatobacter	
ASV634	Bacteria	Proteobacter	Gammaprote	Pseudomona	Pseudomona	Pseudomona	psychrophila
ASV635	Bacteria	Proteobacter	Alphaproteol	Rhizobiales	Rhizobiaceae	Allorhizobium-Neorhizobiu	
ASV636	Bacteria	Proteobacter	Alphaproteol	Rhizobiales	Rhizobiales Ir	Phreatobacter	
ASV637	Bacteria	Proteobacter	Alphaproteol	Rhizobiales	Devosiaceae	Devosia	
ASV638	Bacteria	Actinobacteri	Actinobacteri	Micrococcale	Microbacteria	Microbacterium	
ASV639	Bacteria	Firmicutes	Bacilli	Lactobacillale	Enterococcac	Enterococcus	faecalis
ASV640	Bacteria	Actinobacteri	Actinobacteri	Corynebacter	Corynebacter	Corynebacter	vitaeruminis
ASV641	Bacteria	Proteobacter	Alphaproteol	Sphingomona	Sphingomona	Novosphingobium	
ASV642	Bacteria	Proteobacter	Alphaproteol	Sphingomona	Sphingomona	Novosphingobium	
ASV643	Bacteria	Proteobacter	Gammaprote	Enterobacter	Enterobacter	Klebsiella	variicola
ASV644	Bacteria	Firmicutes	Bacilli	Lactobacillale	Streptococca	Streptococcu	oralis
ASV645	Bacteria	Proteobacter	Gammaprote	Xanthomona	Xanthomona	Luteimonas	
ASV646	Bacteria	Proteobacter	Gammaprote	Enterobacter	Yersiniaceae	Rahnella1	
ASV647	Bacteria	Proteobacter	Gammaprote	Pseudomona	Moraxellacea	Acinetobacte	johnsonii
ASV648	Bacteria	Bacteroidota	Bacteroidia	Cytophagales	Cytophagacea	Siphonobacte	aquaeclarae
ASV649	Bacteria	Firmicutes	Bacilli	Lactobacillale	Streptococca	Streptococcu	oralis

ASV650	Bacteria						
ASV651	Bacteria	Acidobacterio	Blastocatellia	Blastocatellal	Blastocatellaceae		
ASV652	Bacteria	Proteobacter	Alphaproteob	Caulobactera	Caulobactera	Asticcacaulis	excentricus
ASV653	Bacteria	Bacteroidota	Bacteroidia	Cytophagales	Spirosomacea	Pseudarcicell	hirudinis
ASV654	Bacteria	Proteobacter	Gammaprote	Enterobacter	Morganellace	Morganella	morganii
ASV655	Bacteria	Firmicutes	Bacilli	Paenibacillale	Paenibacillac	Paenibacillus	xylanexedens
ASV656	Bacteria	Proteobacter	Gammaprote	Enterobacter	Enterobacteriaceae		
ASV657	Bacteria	Actinobacteri	Actinobacteri	Propionibacte	Propionibacte	Cutibacterium	acnes
ASV658	Bacteria	Firmicutes	Bacilli	Lactobacillale	Streptococca	Streptococcu	oralis
ASV659	Bacteria	Proteobacter	Alphaproteob	Sphingomona	Sphingomona	Novosphingobium	
ASV660	Bacteria	Proteobacter	Gammaprote	Burkholderial	Comamonada	Delftia	acidovorans
ASV661	Bacteria	Proteobacter	Gammaprote	Enterobacter	Enterobacter	Klebsiella	quasipneumo
ASV662	Bacteria	Proteobacter	Gammaprote	Pseudomona	Moraxellacea	Acinetobacter	
ASV663	Bacteria	Proteobacter	Gammaprote	Burkholderial	Oxalobactera	Herbaspirillur	huttiense
ASV664	Bacteria	Firmicutes	Bacilli	Staphylococc	Staphylococc	Staphylococcus	
ASV665	Bacteria	Proteobacter	Gammaprote	Burkholderial	Comamonada	Variovorax	
ASV666	Bacteria	Proteobacter	Gammaprote	Enterobacter	Enterobacter	Enterobacter	
ASV667	Bacteria	Proteobacter	Alphaproteob	Rhizobiales	Rhizobiales Ir	Alsobacter	
ASV668	Bacteria	Proteobacter	Alphaproteob	Caulobactera	Caulobactera	Phenylobacterium	
ASV669	Bacteria						
ASV670	Bacteria	Proteobacter	Gammaprote	Burkholderial	Comamonada	Variovorax	
ASV671	Bacteria	Bacteroidota	Bacteroidia	Sphingobacte	Sphingobacte	Pedobacter	solisilvae
ASV672	Bacteria	Actinobacteri	Actinobacteri	Corynebacter	Corynebacter	Corynebacter	ureicelerivora
ASV673	Bacteria	Proteobacter	Alphaproteob	Sphingomona	Sphingomona	Sphingobium	lactosutens
ASV674	Bacteria	Proteobacter	Alphaproteob	Sphingomona	Sphingomona	Sphingobium	lactosutens
ASV675	Bacteria	Firmicutes	Clostridia	Peptostrepto	Family XI	Fenollaria	
ASV676	Bacteria	Proteobacter	Gammaprote	Xanthomona	Rhodanobact	Frateuria	aurantia
ASV677	Bacteria	Bacteroidota	Bacteroidia	Chitinophaga	Chitinophaga	Rurimicrobium	
ASV678	Bacteria	Actinobacteri	Actinobacteri	Micrococcale	Microbacteria	Microbacteriu	oxydans
ASV679	Bacteria	Proteobacter	Alphaproteob	Acetobactera	Acetobactera	Asaia	krungthepens
ASV680	Bacteria	Proteobacter	Alphaproteob	Rhizobiales	Rhizobiaceae		
ASV681	Bacteria	Firmicutes	Clostridia	Peptostrepto	Family XI	Fenollaria	
ASV682	Bacteria	Proteobacter	Gammaprote	Enterobacter	Shewanellace	Shewanella	xiamenensis
ASV683	Bacteria	Proteobacter	Gammaprote	Burkholderial	Comamonada	Variovorax	
ASV684	Bacteria	Proteobacter	Gammaprote	Xanthomona	Rhodanobact	Frateuria	aurantia
ASV685	Bacteria	Proteobacter	Gammaprote	Enterobacter	Enterobacteriaceae		
ASV686	Bacteria	Actinobacteri	Actinobacteri	Micrococcale	Microbacteria	Microbacteriu	foliorum
ASV687	Bacteria	Bacteroidota	Bacteroidia	Chitinophaga	Chitinophaga	Rurimicrobium	
ASV688	Bacteria	Proteobacter	Gammaprote	Enterobacter	Enterobacter	Klebsiella	variicola
ASV689	Bacteria	Verrucomicro	Verrucomicro	Chthoniobact	Terrimicrobia	Terrimicrobium	
ASV690	Bacteria	Proteobacter	Gammaprote	Burkholderial	Comamonada	Pelomonas	
ASV691	Bacteria	Proteobacter	Gammaprote	Burkholderial	Oxalobactera	Duganella	
ASV692	Bacteria	Proteobacter	Alphaproteob	Rhizobiales	Rhizobiaceae	Allorhizobium-Neorhizobiu	
ASV693	Bacteria	Proteobacter	Gammaprote	Burkholderial	Comamonada	Variovorax	
ASV694	Bacteria	Proteobacter	Gammaprote	Burkholderial	Comamonada	Acidovorax	soli
ASV695	Bacteria	Proteobacter	Gammaprote	Enterobacter	Enterobacteriaceae		
ASV696	Bacteria	Firmicutes	Bacilli	Lactobacillale	Streptococca	Streptococcus	
ASV697	Bacteria	Firmicutes	Bacilli	Lactobacillale	Enterococcac	Enterococcus	faecalis
ASV698	Bacteria	Proteobacter	Gammaprote	Burkholderial	Comamonada	Variovorax	
ASV699	Bacteria	Proteobacter	Gammaprote	Pseudomona	Moraxellacea	Acinetobacte	venetianus

ASV700	Bacteria	Actinobacteri	Actinobacteri	Corynebacter	Corynebacter	Corynebacterium	
ASV701	Bacteria	Proteobacter	Gammaprote	Xanthomona	Rhodanobact	Frateuria	aurantia
ASV702	Bacteria	Proteobacter	Gammaprote	Burkholderial	Comamonada	Variovorax	
ASV703	Bacteria	Bacteroidota	Bacteroidia	Bacteroidetes	VC2.1 Bac22		
ASV704	Bacteria	Proteobacter	Alphaproteol	Rhizobiales	Xanthobacter	Bradyrhizobiu	elkanii
ASV705	Bacteria	Bacteroidota	Bacteroidia	Sphingobacte	Sphingobacte	Sphingobacterium	
ASV706	Bacteria	Proteobacter	Gammaprote	Enterobacter	Enterobacteriaceae		
ASV707	Bacteria	Actinobacteri	Actinobacteri	Micrococcale	Microbacteria	Microbacterium	
ASV708	Bacteria	Actinobacteri	Actinobacteri	Corynebacter	Corynebacter	Corynebacter	ureicelerivora
ASV709	Bacteria	Firmicutes	Negativicutes	Veillonellales	Veillonellacea	Veillonella	parvula
ASV710	Bacteria	Proteobacter	Gammaprote	Enterobacter	Enterobacter	Enterobacter	
ASV711	Bacteria	Bacteroidota	Bacteroidia	Sphingobacte	Sphingobacte	Sphingobacte	siyangense
ASV712	Bacteria	Proteobacter	Gammaprote	Enterobacter	Enterobacter	Klebsiella	oxytoca
ASV713	Bacteria	Actinobacteri	Actinobacteri	Corynebacter	Corynebacter	Lawsonella	clevelandensi
ASV714	Bacteria	Firmicutes	Bacilli	Bacillales	Bacillaceae	Anoxybacillus	flavithermus
ASV715	Bacteria	Firmicutes	Bacilli	Staphylococc	Staphylococc	Staphylococcus	
ASV716	Bacteria	Bacteroidota	Bacteroidia	Sphingobacte	Sphingobacte	Pedobacter	solisilvae
ASV717	Bacteria						
ASV718	Bacteria	Proteobacter	Gammaprote	Enterobacter	Enterobacteriaceae		
ASV719	Bacteria	Actinobacteri	Actinobacteri	Propionibact	Propionibact	Cutibacterium	acnes
ASV720	Bacteria	Proteobacter	Gammaprote	Pseudomona	Moraxellacea	Acinetobacte	soli
ASV721	Bacteria	Proteobacter	Gammaprote	Enterobacter	Enterobacteriaceae		
ASV722	Bacteria	Proteobacter	Gammaprote	Enterobacter	Enterobacter	Klebsiella	
ASV723	Bacteria	Proteobacter	Gammaprote	Enterobacter	Enterobacteriaceae		
ASV724	Bacteria	Firmicutes	Clostridia	Peptostrepto	Family XI	Fenollaria	
ASV725	Bacteria	Proteobacter	Alphaproteol	Rhodobactera	Rhodobactera	Paracoccus	yeei
ASV726	Bacteria	Proteobacter	Gammaprote	Enterobacter	Enterobacter	Klebsiella	
ASV727	Bacteria	Proteobacter	Gammaprote	Burkholderial	Oxalobactera	Undibacteriu	squillarum
ASV728	Bacteria	Proteobacter	Alphaproteol	Azospirillales	Azospirillacea	Azospirillum	
ASV729	Bacteria	Acidobacteri	Blastocatellia	Blastocatellal	Blastocatellaceae		
ASV730	Bacteria	Proteobacter	Alphaproteol	Azospirillales	Azospirillacea	Azospirillum	
ASV731	Bacteria	Proteobacter	Gammaprote	Enterobacter	Enterobacter	Enterobacter	
ASV732	Bacteria	Proteobacter	Gammaprote	Burkholderial	Alcaligenacea	Achromobact	piechaudii
ASV733	Bacteria	Proteobacter	Alphaproteol	Rhizobiales	Rhizobiaceae	Brucella	melitensis
ASV734	Bacteria	Bacteroidota	Bacteroidia	Bacteroidetes	VC2.1 Bac22		
ASV735	Bacteria	Proteobacter	Gammaprote	Xanthomona	Rhodanobact	Frateuria	aurantia
ASV736	Bacteria	Firmicutes	Bacilli	Lactobacillale	Listeriaceae	Listeria	
ASV737	Bacteria	Bacteroidota	Bacteroidia	Cytophagales			
ASV738	Bacteria	Proteobacter	Alphaproteol	Azospirillales	Azospirillacea	Azospirillum	
ASV739	Bacteria	Proteobacter	Alphaproteol	Rhizobiales	Xanthobacter	Rhodopseudomonas	
ASV740	Bacteria	Armatimonac	Armatimonac	Armatimonac	Armatimonac	Armatimonas	
ASV741	Bacteria	Proteobacter	Gammaprote	Burkholderial	Oxalobactera	Undibacteriu	squillarum
ASV742	Bacteria	Proteobacter	Gammaprote	Enterobacter	Enterobacteriaceae		
ASV743	Bacteria	Proteobacter	Gammaprote	Burkholderial	Comamonada	Variovorax	
ASV744	Bacteria	Proteobacter	Alphaproteol	Rhizobiales	Rhizobiaceae	Allorhizobium-Neorhizobiu	
ASV745	Bacteria	Bacteroidota	Bacteroidia	Bacteroidetes	VC2.1 Bac22		
ASV746	Bacteria	Proteobacteria					
ASV747	Bacteria	Firmicutes	Bacilli	Lactobacillale	Streptococca	Streptococcu	salivarius
ASV748	Bacteria	Proteobacter	Gammaprote	Xanthomona	Rhodanobact	Frateuria	aurantia
ASV749	Bacteria	Actinobacteri	Actinobacteri	Propionibact	Propionibact	Cutibacterium	acnes

ASV750	Bacteria	Proteobacter	Gammaprote	Enterobacter	Enterobacter	Klebsiella	quasipneumo
ASV751	Bacteria	Bacteroidota	Bacteroidia	Flavobacteria	Weeksellacea	Cloacibacterium	
ASV752	Bacteria	Actinobacteri	Actinobacteri	Corynebacter	Corynebacter	Corynebacter	coyleae
ASV753	Bacteria	Proteobacter	Gammaprote	Burkholderial	Comamonada	Variovorax	
ASV754	Bacteria	Proteobacter	Gammaprote	Enterobacterales			
ASV755	Bacteria	Bacteroidota	Bacteroidia	Bacteroidetes	VC2.1 Bac22		
ASV756	Bacteria	Firmicutes	Bacilli	Bacillales	Bacillaceae	Anoxybacillus	
ASV757	Bacteria	Proteobacter	Gammaprote	Burkholderial	Comamonada	Variovorax	paradoxus
ASV758	Bacteria	Firmicutes	Bacilli	Staphylococc	Staphylococc	Staphylococc	argenteus
ASV759	Bacteria	Deinococcota	Deinococci	Deinococcale	Deinococcace	Deinococcus	reticulitermiti
ASV760	Bacteria	Bacteroidota	Bacteroidia	Cytophagales	Cytophagace	Siphonobacte	aquaeclarae
ASV761	Bacteria	Proteobacter	Alphaproteob	Rhizobiales	Rhizobiaceae	Allorhizobium-Neorhizobiu	
ASV762	Bacteria	Firmicutes	Bacilli	Lactobacillale	Enterococcac	Enterococcus	casseliflavus
ASV763	Bacteria	Proteobacter	Gammaprote	Pseudomona	Moraxellacea	Acinetobacte	johnsonii
ASV764	Bacteria	Bacteroidota	Bacteroidia	Bacteroidales	Dysgonomon	Proteiniphilum	
ASV765	Bacteria	Bacteroidota	Bacteroidia	Flavobacteria	Weeksellacea	Cloacibacterium	
ASV766	Bacteria	Bacteroidota	Bacteroidia	Cytophagales	Spirosomace	Pseudarcicell	hirudinis
ASV767	Bacteria	Armatimonac	Armatimonac	Armatimonadales			
ASV768	Bacteria	Proteobacter	Gammaprote	Pseudomona	Moraxellacea	Acinetobacte	baylyi
ASV769	Bacteria	Proteobacter	Gammaprote	Enterobacter	Yersiniaceae	Rahnella1	
ASV770	Bacteria	Actinobacteri	Actinobacteri	Corynebacter	Corynebacter	Corynebacterium	
ASV771	Bacteria	Proteobacter	Gammaprote	Burkholderial	Oxalobactera	Herbaspirillur	huttiense
ASV772	Bacteria	Proteobacter	Alphaproteob	Rhizobiales	Xanthobacter	Bradyrhizobiu	elkanii
ASV773	Bacteria	Bacteroidota	Bacteroidia	Cytophagales	Cytophagace	Siphonobacte	aquaeclarae
ASV774	Bacteria	Proteobacter	Alphaproteob	Sphingomona	Sphingomona	Sphingomona	leidyi
ASV775	Bacteria	Proteobacter	Gammaprote	Enterobacter	Enterobacter	Klebsiella	
ASV776	Bacteria	Proteobacter	Alphaproteob	Azospirillales	Azospirillacea	Azospirillum	lipoferum
ASV777	Bacteria						
ASV778	Bacteria	Firmicutes	Bacilli	Staphylococc	Staphylococc	Staphylococc	caprae
ASV779	Bacteria	Proteobacter	Gammaprote	Enterobacter	Enterobacteriaceae		
ASV780	Bacteria	Actinobacteri	Actinobacteri	Micrococcale	Microbacteri	Microbacterium	
ASV781	Bacteria	Proteobacter	Gammaprote	Pseudomona	Moraxellacea	Acinetobacter	
ASV782	Bacteria	Proteobacter	Gammaprote	Burkholderial	Alcaligenacea	Achromobact	piechaudii
ASV783	Bacteria	Proteobacter	Gammaprote	Enterobacter	Enterobacteriaceae		
ASV784	Eukaryota						
ASV785	Bacteria	Firmicutes	Clostridia	Peptostrepto	Family XI	Fenollaria	
ASV786	Eukaryota						
ASV787	Bacteria	Proteobacter	Gammaprote	Salinisphaera	Solimonadac	Nevskia	
ASV788	Bacteria	Proteobacter	Alphaproteob	Rhodobacter	Rhodobacter	Paracoccus	yeei
ASV789	Bacteria	Actinobacteri	Actinobacteri	Corynebacter	Corynebacter	Corynebacter	coyleae
ASV790	Bacteria	Actinobacteri	Actinobacteri	Micrococcale	Microbacteri	Microbacterium	
ASV791	Bacteria	Proteobacter	Gammaprote	Enterobacter	Enterobacteriaceae		
ASV792	Bacteria	Proteobacter	Alphaproteob	Rhizobiales	Xanthobacter	Rhodopseudomonas	
ASV793	Bacteria	Proteobacter	Gammaprote	Pseudomona	Moraxellacea	Acinetobacte	venetianus
ASV794	Bacteria	Firmicutes	Bacilli	Bacillales	Bacillaceae	Anoxybacillus	flavithermus
ASV795	Bacteria	Proteobacter	Alphaproteob	Rhizobiales	Rhizobiaceae	Brucella	
ASV796	Bacteria	Proteobacter	Gammaprote	Burkholderial	Alcaligenacea	Achromobact	piechaudii
ASV797	Bacteria	Proteobacter	Gammaprote	Enterobacter	Enterobacter	Klebsiella	quasipneumo
ASV798	Bacteria	Bacteroidota	Bacteroidia	Cytophagales	Spirosomace	Pseudarcicell	hirudinis
ASV799	Bacteria	Actinobacteri	Actinobacteri	Micrococcale	Microbacteri	Microbacterium	

ASV800	Bacteria	Proteobacter	Gammaprote	Enterobacter	Enterobacteriaceae		
ASV801	Bacteria	Bacteroidota	Bacteroidia	Sphingobacte	Sphingobacte	Sphingobacte	siyangense
ASV802	Bacteria	Proteobacter	Gammaprote	Burkholderial	Oxalobactera	Massilia	
ASV803							
ASV804	Bacteria	Proteobacter	Gammaprote	Pseudomona	Pseudomona	Pseudomonas	
ASV805	Bacteria	Bacteroidota	Bacteroidia	Flavobacteria	Flavobacteria	Flavobacterium	
ASV806	Bacteria	Proteobacter	Gammaprote	Enterobacter	Enterobacteriaceae		
ASV807	Bacteria	Proteobacter	Gammaprote	Enterobacter	Enterobacter	Enterobacter	
ASV808	Bacteria	Proteobacter	Gammaprote	Pseudomona	Moraxellacea	Acinetobacter	
ASV809	Bacteria	Bacteroidota	Bacteroidia	Sphingobacte	Sphingobacte	Sphingobacterium	
ASV810	Eukaryota						
ASV811	Bacteria	Patescibacter	Saccharimona	Saccharimonadales			
ASV812	Bacteria	Firmicutes	Bacilli	Lactobacillale	Lactobacillac	Limosilactobacillus	
ASV813	Bacteria	Proteobacter	Alphaproteol	Rhizobiales	Rhizobiaceae		
ASV814	Bacteria	Proteobacter	Gammaprote	Pseudomona	Moraxellacea	Acinetobacte	baylyi
ASV815	Bacteria	Firmicutes	Clostridia	Peptostrepto	Family XI	Anaerococcus	
ASV816	Bacteria	Proteobacter	Gammaprote	Pseudomona	Pseudomona	Pseudomonas	
ASV817	Bacteria	Bacteroidota	Bacteroidia	Cytophagales	Spirosomace	Pseudarcicell	hirudinis
ASV818	Bacteria	Proteobacter	Gammaprote	Burkholderial	Alcaligenacea	Achromobact	piechaudii
ASV819	Bacteria	Proteobacter	Gammaprote	Enterobacter	Enterobacter	Klebsiella	
ASV820	Bacteria	Firmicutes	Clostridia	Peptostrepto	Family XI	Peptoniphilus	grossensis
ASV821	Bacteria	Proteobacter	Gammaprote	Enterobacter	Enterobacter	Escherichia-Shigella	
ASV822	Bacteria	Firmicutes	Clostridia	Peptostrepto	Family XI	Anaerococcus	
ASV823	Bacteria	Proteobacter	Alphaproteol	Rhizobiales	Rhizobiaceae	Allorhizobium-Neorhizobiu	
ASV824	Bacteria	Actinobacteri	Actinobacteri	Corynebacter	Corynebacter	Corynebacterium	
ASV825	Bacteria	Bacteroidota	Bacteroidia	Flavobacteria	Flavobacteria	Flavobacterium	
ASV826	Bacteria	Firmicutes	Negativicutes	Veillonellales	Veillonellace	Veillonella	atypica
ASV827	Bacteria	Proteobacter	Gammaprote	Pseudomona	Pseudomona	Pseudomona	psychrophila
ASV828	Bacteria	Actinobacteri	Actinobacteri	Corynebacter	Corynebacter	Corynebacterium	
ASV829	Bacteria	Proteobacter	Alphaproteol	Rhodobacter	Rhodobacter	Paracoccus	yeei
ASV830	Bacteria	Proteobacter	Alphaproteol	Rhizobiales	Beijerinckiace	alphaI cluster	
ASV831	Bacteria	Proteobacter	Gammaprote	Enterobacter	Enterobacteriaceae		
ASV832	Bacteria	Bacteroidota	Bacteroidia	Bacteroidales	Dysgonomon	Petrimonas	
ASV833	Bacteria						
ASV834	Bacteria	Bacteroidota	Bacteroidia	Sphingobacte	Sphingobacte	Sphingobacte	thermophilun
ASV835	Bacteria						
ASV836	Bacteria	Proteobacter	Gammaprote	Gammaprote	Unknown Far	Acidibacter	
ASV837	Eukaryota						
ASV838	Bacteria	Proteobacter	Gammaprote	Enterobacter	Enterobacter	Klebsiella	quasipneumo
ASV839	Bacteria	Proteobacter	Gammaprote	Enterobacter	Enterobacteriaceae		
ASV840	Bacteria						
ASV841	Bacteria	Proteobacter	Gammaprote	Enterobacter	Enterobacter	Klebsiella	oxytoca
ASV842	Eukaryota						
ASV843	Bacteria	Firmicutes	Bacilli	Lactobacillale	Lactobacillac	Lactobacillus	iners
ASV844	Bacteria	Actinobacteri	Actinobacteri	Corynebacter	Mycobacteria	Mycobacterium	
ASV845	Bacteria	Bacteroidota	Bacteroidia	Sphingobacte	Sphingobacte	Sphingobacte	multivorum
ASV846	Bacteria						
ASV847	Bacteria	Proteobacter	Alphaproteol	Rhizobiales	Xanthobacter	Bradyrhizobiu	elkanii
ASV848	Bacteria	Proteobacter	Gammaprote	Enterobacter	Enterobacter	Klebsiella	variicola
ASV849	Bacteria	Actinobacteri	Actinobacteri	Propionibact	Propionibact	Cutibacterium	acnes

ASV850	Bacteria	Bacteroidota	Bacteroidia	Flavobacteria	Weeksellacea	Cloacibacterium	
ASV851	Bacteria	Proteobacter	Gammaprote	Enterobacter	Enterobacteriaceae		
ASV852	Bacteria	Firmicutes	Negativicutes	Veillonellales	Veillonellacea	Veillonella	parvula
ASV853	Bacteria	Actinobacteri	Actinobacteri	Corynebacter	Corynebacter	Corynebacter	jeikeium
ASV854	Bacteria	Firmicutes	Bacilli	Staphylococc	Staphylococc	Staphylococcus	
ASV855	Bacteria						
ASV856	Bacteria	Proteobacter	Gammaprote	Enterobacter	Enterobacter	Klebsiella	oxytoca
ASV857	Bacteria	Actinobacteri	Actinobacteri	Micrococcale	Micrococcace	Glutamicibac	arilaitensis
ASV858	Bacteria	Proteobacter	Gammaprote	Pseudomona	Pseudomona	Pseudomona	psychrophila
ASV859	Bacteria	Actinobacteri	Actinobacteri	Corynebacter	Corynebacter	Corynebacter	aurimucosum
ASV860	Bacteria	Actinobacteri	Actinobacteri	Micrococcale	Microbacteri	Microbacterium	
ASV861	Bacteria	Firmicutes	Bacilli	Bacillales	Bacillaceae	Geobacillus	
ASV862	Bacteria	Firmicutes	Bacilli	Lactobacillale	Streptococca	Lactococcus	lactis
ASV863	Bacteria	Firmicutes	Clostridia	Peptostrepto	Family XI	Fenollaria	
ASV864	Bacteria	Proteobacter	Gammaprote	Pseudomona	Pseudomona	Pseudomona	gessardii
ASV865	Bacteria	Actinobacteri	Actinobacteri	Corynebacter	Corynebacter	Corynebacter	urinapleomor
ASV866	Bacteria	Bacteroidota	Bacteroidia	Bacteroidales	Porphyromor	Porphyromor	bennonis
ASV867	Bacteria	Proteobacter	Gammaprote	Enterobacterales			
ASV868	Bacteria	Firmicutes	Bacilli	Lactobacillale	Enterococcac	Enterococcus	
ASV869	Bacteria	Proteobacter	Alphaproteol	Rhizobiales	Rhizobiaceae	Allorhizobium-Neorhizobiu	
ASV870	Bacteria	Proteobacter	Gammaprote	Burkholderial	Comamonad	Acidovorax	
ASV871	Bacteria	Firmicutes	Bacilli	Lactobacillale	Enterococcac	Enterococcus	
ASV872	Bacteria	Proteobacter	Alphaproteol	Rhizobiales	Beijerinckiace	alphaI cluster	
ASV873	Bacteria	Firmicutes	Bacilli	Staphylococc	Staphylococc	Staphylococcus	
ASV874	Bacteria	Actinobacteri	Actinobacteri	Corynebacter	Corynebacter	Corynebacter	tuberculostea
ASV875	Bacteria	Actinobacteri	Actinobacteri	Corynebacter	Corynebacter	Corynebacter	pseudogenita
ASV876	Bacteria	Firmicutes	Bacilli	Lactobacillale	Enterococcac	Enterococcus	moraviensis
ASV877	Bacteria	Bacteroidota	Bacteroidia	Flavobacteria	Weeksellacea	Cloacibacterium	
ASV878	Bacteria	Actinobacteri	Actinobacteri	Corynebacter	Corynebacter	Corynebacter	tuberculostea
ASV879	Bacteria	Proteobacter	Alphaproteol	Sphingomona	Sphingomonadaceae		
ASV880	Bacteria	Proteobacter	Gammaprote	Enterobacter	Enterobacter	Klebsiella	
ASV881	Bacteria	Bacteroidota	Bacteroidia	Bacteroidales	Porphyromor	Porphyromonas	
ASV882	Bacteria	Actinobacteri	Actinobacteri	Corynebacter	Corynebacter	Corynebacter	ureicelerivora
ASV883	Bacteria	Firmicutes	Bacilli	Staphylococc	Staphylococc	Staphylococc	aureus
ASV884	Bacteria	Firmicutes	Clostridia	Peptostrepto	Family XI	Anaerococcus	
ASV885	Bacteria	Proteobacter	Gammaprote	Enterobacter	Enterobacter	Escherichia-Shigella	
ASV886	Bacteria	Proteobacter	Gammaprote	Pseudomona	Pseudomona	Pseudomona	psychrophila
ASV887	Bacteria	Bacteroidota	Bacteroidia	Flavobacteria	Weeksellacea	Moheibacter	
ASV888	Bacteria	Firmicutes	Bacilli	Lactobacillale	Streptococca	Streptococcus	
ASV889	Bacteria	Proteobacter	Alphaproteol	Sphingomona	Sphingomona	Novosphingobium	
ASV890	Bacteria	Bacteroidota	Bacteroidia	Chitinophaga	Chitinophaga	Taibaiella	
ASV891	Bacteria	Proteobacter	Gammaprote	Pseudomona	Moraxellacea	Acinetobacte	soli
ASV892	Bacteria	Proteobacter	Gammaprote	Pseudomona	Pseudomona	Pseudomona	parafulva
ASV893	Bacteria	Proteobacter	Gammaprote	Enterobacter	Yersiniaceae	Serratia	
ASV894	Bacteria	Firmicutes	Clostridia	Peptostrepto	Family XI	Anaerococcus	
ASV895	Bacteria	Firmicutes	Bacilli	Staphylococc	Staphylococc	Staphylococcus	
ASV896	Bacteria	Proteobacter	Gammaprote	Pseudomona	Pseudomona	Pseudomona	psychrophila
ASV897	Bacteria	Proteobacter	Gammaprote	Enterobacter	Enterobacter	Escherichia-Shigella	
ASV898	Bacteria	Firmicutes	Bacilli	Lactobacillale	Streptococca	Streptococcus	
ASV899	Bacteria	Proteobacter	Gammaprote	Pseudomona	Moraxellacea	Acinetobacter	

ASV900	Bacteria	Proteobacter	Alphaproteol	Caulobactera	Caulobactera	Phenylobacterium	
ASV901	Bacteria	Proteobacter	Alphaproteol	Caulobactera	Caulobactera	Caulobacter	vibrioides
ASV902	Bacteria	Proteobacter	Gammaprote	Enterobacter	Enterobacter	Klebsiella	
ASV903	Bacteria	Proteobacter	Gammaprote	Enterobacter	Enterobacter	Escherichia-Shigella	
ASV904	Bacteria	Firmicutes	Negativicutes	Veillonellales	Veillonellacea	Veillonella	
ASV905	Bacteria	Proteobacter	Gammaprote	Enterobacter	Enterobacteriaceae		
ASV906	Bacteria	Firmicutes	Negativicutes	Veillonellales	Veillonellacea	Veillonella	parvula
ASV907	Bacteria	Firmicutes	Bacilli	Staphylococc	Staphylococc	Staphylococcus	
ASV908	Bacteria						
ASV909	Bacteria	Actinobacteri	Actinobacteri	Corynebacter	Corynebacter	Corynebacterium	
ASV910	Bacteria	Firmicutes	Bacilli	Staphylococc	Staphylococc	Staphylococcus	
ASV911	Bacteria	Firmicutes	Bacilli	Staphylococc	Staphylococc	Staphylococcus	
ASV912	Bacteria						
ASV913	Bacteria	Patescibacter	Saccharimona	Saccharimonadales			
ASV914	Bacteria	Proteobacter	Alphaproteol	Sphingomona	Sphingomona	Novosphingo	sediminicola
ASV915	Bacteria	Bacteroidota	Bacteroidia	Bacteroidales	Porphyromor	Porphyromor	bennonis
ASV916	Bacteria	Proteobacter	Gammaprote	Enterobacter	Enterobacteriaceae		
ASV917	Bacteria						
ASV918	Bacteria	Proteobacter	Gammaprote	Pseudomona	Pseudomona	Pseudomonas	
ASV919	Bacteria	Firmicutes	Negativicutes	Veillonellales	Veillonellacea	Veillonella	parvula
ASV920	Bacteria	Proteobacter	Gammaprote	Enterobacter	Enterobacter	Enterobacter	
ASV921	Bacteria	Bacteroidota	Bacteroidia	Sphingobacte	Sphingobacte	Sphingobacte	thermophilum
ASV922	Bacteria	Firmicutes	Clostridia	Peptostrepto	Family XI	Finegoldia	magna
ASV923	Bacteria						
ASV924	Bacteria	Proteobacter	Gammaprote	Enterobacter	Enterobacteriaceae		
ASV925	Bacteria	Firmicutes	Bacilli	Staphylococc	Staphylococc	Staphylococc	aureus
ASV926	Bacteria	Actinobacteri	Actinobacteri	Corynebacter	Corynebacter	Corynebacterium	
ASV927	Bacteria						
ASV928	Bacteria	Proteobacter	Gammaprote	Pseudomona	Moraxellacea	Acinetobacte	baylyi
ASV929	Bacteria	Proteobacter	Alphaproteol	Sphingomona	Sphingomona	Sphingomonas	
ASV930	Bacteria	Firmicutes	Clostridia	Peptostrepto	Family XI	Anaerococcus	
ASV931	Bacteria	Proteobacter	Gammaprote	Pseudomona	Pseudomona	Pseudomonas	
ASV932	Bacteria	Proteobacter	Alphaproteol	Rhizobiales	Rhizobiales Ir	Phreatobacter	
ASV933	Bacteria	Bacteroidota	Bacteroidia	Sphingobacte	Sphingobacte	Sphingobacte	multivorum
ASV934	Bacteria	Proteobacter	Gammaprote	Enterobacter	Enterobacter	Enterobacter	
ASV935	Bacteria	Firmicutes	Clostridia	Peptostrepto	Family XI	Anaerococcus	prevotii
ASV936	Bacteria	Proteobacter	Gammaprote	Burkholderial	Comamonada	Acidovorax	
ASV937	Bacteria						
ASV938	Bacteria	Proteobacter	Gammaprote	Pseudomona	Moraxellacea	Acinetobacte	soli
ASV939	Bacteria	Proteobacter	Gammaprote	Enterobacter	Enterobacter	Escherichia-Shigella	
ASV940	Bacteria	Firmicutes	Bacilli	Staphylococc	Staphylococc	Staphylococcus	
ASV941	Bacteria						
ASV942	Bacteria	Firmicutes	Bacilli	Lactobacillale	Enterococcac	Enterococcus	
ASV943	Bacteria	Proteobacter	Gammaprote	Pseudomona	Moraxellacea	Acinetobacte	baylyi
ASV944	Bacteria						
ASV945	Bacteria	Proteobacter	Alphaproteol	Rhizobiales	Xanthobacter	Bradyrhizobiu	liaoningense
ASV946	Bacteria	Firmicutes	Clostridia	Peptostrepto	Family XI	Peptoniphilus	lacrimalis
ASV947	Bacteria	Firmicutes	Bacilli	Staphylococc	Staphylococc	Staphylococcus	
ASV948	Bacteria	Proteobacter	Alphaproteol	Rhizobiales	Xanthobacter	Rhodopseudomonas	
ASV949	Bacteria	Proteobacter	Alphaproteol	Rhizobiales	Rhizobiaceae	Allorhizobium-Neorhizobium	

ASV950	Bacteria	Firmicutes	Negativicutes	Veillonellales	Veillonellacea	Veillonella	
ASV951	Bacteria	Proteobacter	Gammaprote	Pseudomona	Moraxellacea	Acinetobacte	baylyi
ASV952	Bacteria	Proteobacter	Gammaprote	Pseudomona	Pseudomona	Pseudomona	otitidis
ASV953	Bacteria	Proteobacter	Gammaprote	Pseudomona	Pseudomona	Pseudomonas	
ASV954	Bacteria	Firmicutes	Clostridia	Peptostrepto	Family XI	Anaerococcus	mediterranee
ASV955	Bacteria	Proteobacter	Gammaprote	Burkholderial	Comamonada	Xylophilus	
ASV956	Bacteria	Firmicutes	Clostridia	Peptostrepto	Family XI	Gallicola	
ASV957	Eukaryota						
ASV958	Bacteria						
ASV959	Bacteria	Proteobacter	Gammaprote	Enterobacter	Yersiniaceae	Rahnella1	
ASV960	Bacteria	Firmicutes	Bacilli	Lactobacillale	Lactobacillace	Lactobacillus	iners
ASV961	Bacteria	Proteobacter	Gammaprote	Burkholderial	Comamonada	Acidovorax	
ASV962	Bacteria	Proteobacter	Gammaprote	Burkholderial	Comamonada	Delftia	
ASV963	Bacteria	Verrucomicro	Chlamydiae	Chlamydiales	Parachlamydiaceae		
ASV964	Bacteria	Proteobacter	Gammaprote	Pseudomona	Pseudomona	Pseudomona	fluorescens
ASV965	Bacteria	Actinobacteri	Actinobacteri	Corynebacter	Corynebacter	Lawsonella	clevelandensi
ASV966	Bacteria	Firmicutes	Bacilli	Lactobacillale	Streptococca	Streptococcus	
ASV967	Bacteria	Firmicutes	Bacilli	Bacillales	Bacillaceae	Bacillus	
ASV968	Bacteria	Proteobacter	Gammaprote	Pseudomona	Pseudomona	Pseudomonas	
ASV969	Bacteria	Proteobacter	Alphaproteob	Rhizobiales	Beijerinckiaceae		
ASV970	Bacteria	Proteobacter	Gammaprote	Burkholderial	Comamonadaceae		
ASV971	Bacteria	Actinobacteri	Actinobacteri	Micrococcale	Microbacteria	Leucobacter	
ASV972	Bacteria	Firmicutes	Bacilli	Lactobacillale	Streptococca	Streptococcus	
ASV973	Bacteria	Proteobacter	Alphaproteob	Caulobactera	Caulobactera	Phenylobacterium	
ASV974	Bacteria	Proteobacter	Gammaprote	Enterobacter	Enterobacter	Klebsiella	
ASV975	Bacteria	Proteobacter	Gammaprote	Burkholderial	Nitrosomona	DSSD61	
ASV976	Bacteria	Proteobacter	Gammaprote	Enterobacter	Enterobacteriaceae		
ASV977	Eukaryota						
ASV978	Eukaryota						
ASV979	Bacteria	Proteobacter	Gammaprote	Enterobacter	Enterobacter	Klebsiella	
ASV980	Bacteria	Proteobacter	Gammaprote	Enterobacter	Enterobacter	Klebsiella	
ASV981	Bacteria	Firmicutes	Bacilli	Lactobacillale	Streptococca	Streptococcus	
ASV982	Bacteria	Proteobacter	Alphaproteob	Sphingomona	Sphingomona	Sphingomona	azotifigens
ASV983	Bacteria	Bacteroidota	Bacteroidia	Sphingobacte	Sphingobacte	Sphingobacte	siyangense
ASV984	Bacteria	Proteobacter	Alphaproteob	Rhizobiales	Methyloligellaceae		
ASV985	Bacteria						
ASV986	Bacteria	Firmicutes	Bacilli	Staphylococc	Staphylococc	Staphylococc	aureus
ASV987	Bacteria	Bacteroidota	Bacteroidia	Cytophagales	Spirosomacea	Pseudarcicell	hirudinis
ASV988	Bacteria	Bdellovibrion	Bdellovibrion	Bdellovibrion	Bdellovibrion	Bdellovibrio	
ASV989	Bacteria	Proteobacter	Gammaprote	Burkholderial	Comamonada	Acidovorax	
ASV990	Bacteria	Proteobacter	Alphaproteob	Sphingomona	Sphingomona	Novosphingobium	
ASV991	Bacteria	Proteobacter	Gammaprote	Enterobacter	Enterobacter	Escherichia-Shigella	
ASV992	Bacteria	Firmicutes	Bacilli	Bacillales	Bacillaceae	Bacillus	
ASV993	Bacteria	Proteobacter	Gammaprote	Gammaprote	Unknown Far	Acidibacter	
ASV994	Bacteria	Firmicutes	Clostridia	Peptostrepto	Family XI	Ezakiella	
ASV995	Bacteria	Proteobacter	Gammaprote	Enterobacter	Enterobacter	Klebsiella	
ASV996	Bacteria	Proteobacter	Gammaprote	Burkholderial	Comamonadaceae		
ASV997	Bacteria	Proteobacter	Alphaproteob	Rhizobiales	Xanthobacteraceae		
ASV998	Bacteria	Bacteroidota	Bacteroidia	Chitinophaga	Chitinophaga	Taibaiella	
ASV999	Bacteria	Firmicutes	Bacilli	Staphylococc	Staphylococc	Staphylococcus	

ASV1000	Bacteria	Proteobacter	Gammaprote	Enterobacter	Erwiniaceae	Erwinia	
ASV1001	Bacteria	Firmicutes	Bacilli	Staphylococc	Staphylococc	Staphylococcus	
ASV1002	Bacteria	Proteobacter	Gammaprote	Enterobacter	Enterobacteriaceae		
ASV1003	Bacteria	Proteobacter	Gammaprote	Burkholderial	Comamonadaceae		
ASV1004	Bacteria	Abditibacteri	Abditibacteri	Abditibacteri	Abditibacteri	Abditibacterium	
ASV1005	Bacteria	Proteobacter	Gammaprote	Pseudomona	Moraxellacea	Acinetobacte	baylyi
ASV1006	Bacteria	Proteobacter	Alphaproteol	Sphingomona	Sphingomona	Sphingomonas	
ASV1007	Bacteria	Proteobacter	Gammaprote	Enterobacter	Yersiniaceae	Serratia	
ASV1008	Bacteria						
ASV1009	Bacteria						
ASV1010	Bacteria	Firmicutes	Negativicutes	Veillonellales	Veillonellacea	Veillonella	parvula
ASV1011	Bacteria	Actinobacteri	Actinobacteri	Corynebacter	Corynebacter	Corynebacterium	
ASV1012	Bacteria						
ASV1013	Bacteria						
ASV1014	Bacteria						
ASV1015	Bacteria	Proteobacter	Gammaprote	Enterobacter	Enterobacter	Enterobacter	
ASV1016	Bacteria	Firmicutes	Bacilli	Lactobacillales			
ASV1017	Bacteria	Proteobacter	Gammaprote	Pseudomona	Moraxellacea	Acinetobacte	variabilis
ASV1018	Bacteria	Proteobacter	Gammaprote	Burkholderial	Comamonada	Acidovorax	
ASV1019	Bacteria	Bacteroidota	Bacteroidia	Sphingobacte	Sphingobacte	Sphingobacte	siyangense
ASV1020	Bacteria	Proteobacter	Alphaproteol	Rhodobactera	Rhodobactera	Paracoccus	marinus
ASV1021	Bacteria						
ASV1022	Bacteria						
ASV1023	Bacteria						
ASV1024	Bacteria	Proteobacter	Gammaprote	Enterobacter	Enterobacter	Enterobacter	
ASV1025	Bacteria	Proteobacter	Gammaprote	Enterobacter	Enterobacteriaceae		
ASV1026	Bacteria	Proteobacter	Gammaprote	Enterobacter	Yersiniaceae	Serratia	
ASV1027							
ASV1028	Bacteria	Proteobacter	Alphaproteol	Rickettsiales	Mitochondria		
ASV1029	Bacteria	Proteobacter	Alphaproteol	Sphingomona	Sphingomona	Novosphingobium	
ASV1030	Bacteria	Proteobacter	Gammaprote	Pseudomona	Pseudomona	Pseudomonas	
ASV1031	Bacteria	Proteobacter	Gammaprote	Enterobacter	Yersiniaceae	Rahnella1	
ASV1032	Bacteria	Proteobacter	Gammaprote	Burkholderial	Comamonadaceae		
ASV1033	Eukaryota						
ASV1034	Bacteria	Firmicutes	Bacilli	Lactobacillale	Streptococca	Streptococcus	
ASV1035	Bacteria	Proteobacter	Gammaprote	Pseudomona	Pseudomona	Pseudomonas	
ASV1036	Bacteria						
ASV1037	Bacteria	Proteobacteria					
ASV1038	Eukaryota						
ASV1039	Bacteria	Proteobacter	Gammaprote	Burkholderial	Neisseriaceae		
ASV1040	Bacteria	Proteobacter	Alphaproteol	Reyranellales	Reyranellacea	Reyranella	massiliensis
ASV1041	Bacteria	Proteobacter	Alphaproteol	Rhizobiales	Rhizobiaceae	Pseudochrob	saccharolyticu
ASV1042	Bacteria	Proteobacter	Gammaprote	Enterobacter	Yersiniaceae	Serratia	
ASV1043	Bacteria	Bacteroidota	Bacteroidia	Flavobacteria	Weeksellacea	Cloacibacterium	
ASV1044	Bacteria	Proteobacter	Gammaprote	Gammaprote	Unknown Far	Acidibacter	
ASV1045	Bacteria	Firmicutes	Bacilli	Lactobacillale	Enterococcac	Enterococcus	faecalis
ASV1046	Eukaryota						
ASV1047	Bacteria						
ASV1048	Bacteria	Proteobacter	Gammaprote	Burkholderial	Comamonada	Delftia	
ASV1049	Bacteria	Firmicutes	Bacilli	Lactobacillale	Enterococcac	Enterococcus	

ASV1050	Bacteria	Proteobacter	Gammaprote	Enterobacter	Yersiniaceae	Serratia	
ASV1051	Bacteria	Proteobacter	Gammaprote	Enterobacter	Enterobacteriaceae		
ASV1052	Bacteria	Proteobacter	Gammaprote	Enterobacter	Enterobacteriaceae		
ASV1053	Bacteria	Actinobacteri	Actinobacteri	Corynebacter	Corynebacter	Corynebacter	pilbarense
ASV1054	Eukaryota						
ASV1055	Bacteria	Actinobacteri	Actinobacteri	Corynebacter	Corynebacter	Corynebacterium	
ASV1056	Bacteria	Proteobacter	Alphaproteob	Acetobactera	Acetobactera	Gluconobacter	
ASV1057	Bacteria	Proteobacter	Gammaprote	Pseudomona	Pseudomona	Pseudomonas	
ASV1058	Bacteria	Proteobacter	Gammaprote	Enterobacter	Enterobacter	Enterobacter	
ASV1059	Bacteria	Proteobacter	Gammaprote	Enterobacter	Enterobacter	Klebsiella	michiganensis
ASV1060	Bacteria	Firmicutes	Bacilli	Lactobacillale	Lactobacillaceae		
ASV1061	Bacteria	Proteobacter	Gammaprote	Burkholderial	Oxalobactera	Massilia	
ASV1062	Bacteria	Bacteroidota	Bacteroidia	Flavobacteria	Weeksellacea	Cloacibacterium	
ASV1063	Bacteria	Proteobacter	Gammaprote	Pseudomona	Pseudomona	Pseudomonas	
ASV1064	Bacteria	Proteobacter	Alphaproteob	Rhodobactera	Rhodobactera	Paracoccus	
ASV1065	Bacteria	Proteobacter	Gammaprote	Enterobacter	Aeromonada	Aeromonas	
ASV1066	Bacteria						
ASV1067	Bacteria						
ASV1068	Bacteria	Firmicutes	Bacilli	Staphylococc	Staphylococc	Staphylococcus	
ASV1069	Bacteria	Firmicutes	Bacilli	Staphylococc	Gemellaceae	Gemella	haemolysans
ASV1070	Bacteria	Proteobacter	Gammaprote	Enterobacter	Enterobacteriaceae		
ASV1071	Bacteria	Proteobacter	Gammaprote	Enterobacter	Yersiniaceae	Yersinia	
ASV1072	Bacteria	Firmicutes	Bacilli	Lactobacillale	Lactobacillaceae		
ASV1073	Bacteria	Actinobacteri	Actinobacteri	Corynebacter	Corynebacter	Corynebacterium	
ASV1074	Bacteria	Proteobacter	Gammaprote	Burkholderial	Comamonada	Curvibacter	
ASV1075	Bacteria	Proteobacter	Gammaprote	Enterobacter	Enterobacter	Klebsiella	
ASV1076	Bacteria	Proteobacter	Gammaprote	Burkholderial	Rhodocyclace	Thauera	
ASV1077	Bacteria	Firmicutes	Bacilli	Staphylococc	Staphylococc	Staphylococc	devriesei
ASV1078	Bacteria	Proteobacter	Gammaprote	Enterobacter	Yersiniaceae	Yersinia	pekkanenii
ASV1079	Bacteria						
ASV1080	Bacteria	Proteobacter	Gammaprote	Burkholderial	Comamonadaceae		
ASV1081	Bacteria	Proteobacter	Gammaprote	Enterobacter	Enterobacteriaceae		
ASV1082	Eukaryota						
ASV1083	Bacteria	Proteobacter	Gammaprote	Enterobacter	Enterobacter	Escherichia-Shigella	
ASV1084	Bacteria	Proteobacter	Gammaprote	Enterobacter	Enterobacter	Enterobacter	
ASV1085	Bacteria	Firmicutes	Bacilli	Bacillales	Bacillaceae	Bacillus	
ASV1086	Bacteria	Proteobacter	Gammaprote	Enterobacter	Enterobacteriaceae		
ASV1087							
ASV1088	Bacteria						
ASV1089	Bacteria	Proteobacter	Gammaprote	Enterobacter	Yersiniaceae	Rahnella1	
ASV1090	Bacteria	Firmicutes	Negativicutes	Veillonellales	Veillonellacea	Veillonella	parvula
ASV1091	Bacteria	Firmicutes	Bacilli	Lactobacillale	Listeriaceae	Listeria	marthii
ASV1092	Bacteria	Proteobacter	Gammaprote	Enterobacter	Enterobacteriaceae		
ASV1093	Bacteria	Proteobacter	Gammaprote	Enterobacter	Yersiniaceae	Yersinia	
ASV1094	Bacteria	Proteobacter	Gammaprote	Enterobacter	Aeromonada	Aeromonas	encheleia
ASV1095	Eukaryota						
ASV1096	Bacteria						
ASV1097	Bacteria	Proteobacter	Gammaprote	Enterobacter	Enterobacter	Escherichia-Shigella	
ASV1098	Bacteria	Bacteroidota	Bacteroidia	Cytophagales	Spirosomacea	Pseudarcicell	hirudinis
ASV1099	Bacteria	Proteobacter	Gammaprote	Enterobacter	Yersiniaceae	Rahnella1	

ASV1100	Bacteria	Proteobacter	Gammaprote	Pseudomona	Pseudomona	Pseudomonas	
ASV1101	Bacteria	Firmicutes	Bacilli	Staphylococc	Staphylococc	Staphylococcus	
ASV1102	Bacteria	Firmicutes	Bacilli	Lactobacillale	Lactobacillac	Limosilactobacillus	
ASV1103	Bacteria	Firmicutes	Bacilli	Lactobacillale	Lactobacillaceae		
ASV1104	Bacteria	Proteobacter	Gammaprote	Enterobacter	Yersiniaceae	Serratia	nematodiphil
ASV1105	Bacteria	Proteobacter	Gammaprote	Pseudomona	Pseudomona	Pseudomonas	
ASV1106	Bacteria	Proteobacter	Alphaproteol	Rhizobiales	Devosiaceae	Devosia	
ASV1107	Bacteria	Proteobacter	Gammaprote	Enterobacter	Enterobacter	Enterobacter	
ASV1108	Bacteria	Proteobacter	Gammaprote	Pseudomona	Pseudomona	Pseudomonas	
ASV1109	Bacteria	Proteobacter	Gammaprote	Enterobacterales			
ASV1110	Bacteria	Bacteroidota	Bacteroidia	Flavobacteria	Weeksellacea	Chryseobacterium	
ASV1111	Bacteria						
ASV1112	Bacteria	Firmicutes	Bacilli	Lactobacillale	Lactobacillac	Limosilactobacillus	
ASV1113	Bacteria	Proteobacter	Gammaprote	Burkholderial	Methylophila	Methylophilus	
ASV1114	Bacteria	Proteobacter	Alphaproteol	Sphingomona	Sphingomona	Sphingomona	paucimobilis
ASV1115	Bacteria	Proteobacter	Alphaproteol	Caulobactera	Caulobactera	Asticcacaulis	excentricus
ASV1116	Bacteria	Proteobacter	Gammaprote	Enterobacterales			
ASV1117	Bacteria	Firmicutes	Bacilli	Bacillales	Bacillaceae	Bacillus	
ASV1118	Bacteria	Actinobacteri	Actinobacteri	Corynebacter	Corynebacter	Corynebacter	pilbarense
ASV1119	Bacteria	Proteobacter	Gammaprote	Pseudomona	Moraxellacea	Acinetobacter	
ASV1120	Bacteria	Proteobacter	Gammaprote	Enterobacter	Enterobacteriaceae		
ASV1121	Bacteria	Proteobacter	Gammaprote	Enterobacter	Enterobacter	Enterobacter	
ASV1122	Bacteria	Proteobacter	Gammaprote	Pseudomona	Pseudomona	Pseudomonas	
ASV1123	Bacteria	Proteobacter	Gammaprote	Enterobacter	Enterobacter	Cedecea	
ASV1124	Bacteria	Proteobacter	Gammaprote	Pseudomona	Pseudomona	Pseudomona	floridensis
ASV1125	Bacteria	Proteobacter	Gammaprote	Enterobacter	Enterobacter	Escherichia-S	fergusonii
ASV1126	Bacteria	Proteobacter	Gammaprote	Enterobacter	Enterobacteriaceae		
ASV1127	Bacteria	Proteobacter	Gammaprote	Pseudomona	Pseudomona	Pseudomonas	
ASV1128	Bacteria	Bacteroidota	Bacteroidia	Flavobacteria	Weeksellacea	Chryseobacte	massiliae
ASV1129	Bacteria	Proteobacter	Gammaprote	Pseudomona	Pseudomona	Pseudomonas	
ASV1130	Bacteria	Actinobacteri	Actinobacteri	Corynebacter	Corynebacter	Corynebacterium	
ASV1131	Bacteria	Deinococcota	Deinococci	Deinococcale	Trueperaceae	Truepera	
ASV1132	Bacteria	Proteobacter	Gammaprote	Pseudomona	Pseudomona	Pseudomonas	
ASV1133	Bacteria	Actinobacteri	Actinobacteri	Corynebacter	Corynebacter	Corynebacterium	
ASV1134	Bacteria						
ASV1135	Bacteria	Proteobacter	Gammaprote	Enterobacter	Yersiniaceae	Yersinia	pekkanenii
ASV1136	Bacteria	Firmicutes	Bacilli	Bacillales	Bacillaceae		
ASV1137	Bacteria	Firmicutes	Bacilli				
ASV1138	Bacteria	Firmicutes	Bacilli	Lactobacillale	Listeriaceae	Listeria	
ASV1139	Bacteria	Proteobacter	Gammaprote	Burkholderial	Comamonada	Ottowia	
ASV1140	Bacteria	Proteobacter	Gammaprote	Pseudomona	Moraxellacea	Acinetobacte	lwoffii
ASV1141	Bacteria	Firmicutes	Bacilli	Paenibacillale	Paenibacillac	Paenibacillus	
ASV1142	Bacteria	Firmicutes	Negativicutes	Veillonellales	Veillonellacea	Veillonella	
ASV1143	Bacteria						
ASV1144	Bacteria	Firmicutes	Bacilli	Bacillales	Bacillaceae	Bacillus	mojavensis
ASV1145	Eukaryota						
ASV1146	Bacteria	Proteobacter	Alphaproteol	Caulobactera	Caulobactera	Phenylobacterium	
ASV1147	Bacteria	Bacteroidota	Bacteroidia	Flavobacteria	Weeksellacea	Chryseobacte	massiliae
ASV1148	Bacteria	Proteobacter	Gammaprote	Enterobacter	Enterobacteriaceae		
ASV1149	Bacteria	Firmicutes	Bacilli	Lactobacillale	Aerococcacea	Facklamia	languida

ASV1150	Bacteria	Firmicutes	Bacilli	Bacillales	Planococcace	Sporosarcina	
ASV1151	Bacteria	Firmicutes	Bacilli				
ASV1152	Bacteria	Firmicutes	Bacilli	Lactobacillale	Lactobacillaceae		
ASV1153	Bacteria	Firmicutes	Bacilli	Lactobacillale	Lactobacillaceae		
ASV1154	Bacteria	Proteobacter	Gammaprote	Enterobacter	Enterobacteriaceae		
ASV1155	Bacteria	Bacteroidota	Bacteroidia	Flavobacteria	Weeksellacea	Elizabethking	bruuniana
ASV1156	Bacteria	Firmicutes	Bacilli	Lactobacillale	Enterococcac	Enterococcus	
ASV1157	Bacteria	Proteobacter	Gammaprote	Enterobacter	Yersiniaceae	Yersinia	pekkanenii
ASV1158							
ASV1159	Bacteria	Firmicutes	Bacilli	Lactobacillale	Streptococca	Streptococcus	
ASV1160	Bacteria						
ASV1161	Bacteria	Firmicutes	Bacilli	Staphylococc	Staphylococc	Staphylococcus	
ASV1162	Bacteria						
ASV1163	Bacteria						
ASV1164	Eukaryota						
ASV1165	Bacteria	Proteobacter	Gammaprote	Enterobacter	Enterobacter	Enterobacter	
ASV1166	Bacteria	Proteobacter	Gammaprote	Enterobacter	Enterobacteriaceae		
ASV1167	Bacteria	Bacteroidota	Bacteroidia	Flavobacteria	Weeksellacea	Elizabethking	anophelis
ASV1168	Bacteria	Firmicutes	Bacilli	Staphylococc	Staphylococc	Staphylococc	devriesei
ASV1169	Bacteria	Firmicutes	Bacilli	Bacillales	Bacillaceae	Bacillus	
ASV1170	Bacteria	Firmicutes	Bacilli	Bacillales	Bacillaceae	Bacillus	intestinalis
ASV1171	Bacteria	Actinobacteri	Actinobacteri	Corynebacter	Corynebacter	Corynebacterium	
ASV1172	Bacteria						
ASV1173	Bacteria	Firmicutes	Bacilli	Lactobacillale	Listeriaceae	Listeria	marthii
ASV1174	Bacteria	Proteobacter	Gammaprote	Pseudomona	Pseudomona	Pseudomonas	
ASV1175	Bacteria	Firmicutes	Bacilli	Lactobacillale	Enterococcac	Enterococcus	
ASV1176	Bacteria	Firmicutes	Bacilli	Lactobacillale	Lactobacillac	Limosilactobacillus	
ASV1177	Bacteria	Firmicutes	Bacilli	Staphylococc	Staphylococc	Staphylococcus	
ASV1178	Bacteria	Firmicutes	Negativicutes	Veillonellales	Veillonellacea	Veillonella	dispar
ASV1179	Bacteria	Firmicutes	Bacilli	Lactobacillale	Listeriaceae	Listeria	marthii
ASV1180	Bacteria	Firmicutes	Bacilli				
ASV1181	Bacteria	Firmicutes	Bacilli	Lactobacillale	Streptococca	Streptococcus	
ASV1182	Bacteria						
ASV1183	Eukaryota						
ASV1184	Bacteria	Proteobacter	Gammaprote	Enterobacter	Yersiniaceae	Yersinia	
ASV1185	Bacteria	Proteobacter	Gammaprote	Enterobacter	Yersiniaceae	Yersinia	pekkanenii
ASV1186	Bacteria	Actinobacteri	Actinobacteri	Micrococcale	Microbacteria	Microbacteri	laevaniformar
ASV1187	Bacteria	Proteobacter	Gammaprote	Enterobacter	Yersiniaceae	Rahnella1	
ASV1188	Bacteria	Proteobacter	Gammaprote	Enterobacter	Yersiniaceae	Yersinia	
ASV1189	Bacteria	Firmicutes	Bacilli	Lactobacillale	Listeriaceae		
ASV1190	Bacteria	Firmicutes	Bacilli	Staphylococc	Staphylococc	Staphylococcus	
ASV1191	Bacteria						
ASV1192	Bacteria	Firmicutes	Bacilli	Lactobacillale	Listeriaceae	Listeria	marthii
ASV1193	Bacteria	Firmicutes	Bacilli	Bacillales	Bacillaceae	Bacillus	mojavensis
ASV1194	Bacteria	Firmicutes	Bacilli	Staphylococc	Staphylococc	Staphylococcus	
ASV1195	Bacteria	Firmicutes	Bacilli	Lactobacillale	Lactobacillaceae		
ASV1196	Bacteria						
ASV1197	Bacteria	Firmicutes	Bacilli	Staphylococc	Staphylococc	Staphylococc	devriesei
ASV1198	Bacteria	Proteobacter	Alphaproteol	Sphingomona	Sphingomona	Novosphingobium	
ASV1199	Bacteria	Firmicutes	Bacilli	Lactobacillale	Enterococcac	Enterococcus	

ASV1200	Bacteria	Proteobacter	Gammaprote	Pseudomona	Moraxellacea	Acinetobacter	
ASV1201	Bacteria	Proteobacter	Gammaprote	Pseudomona	Moraxellaceae		
ASV1202	Bacteria	Firmicutes	Bacilli	Lactobacillale	Streptococca	Streptococcus	
ASV1203	Bacteria	Bacteroidota	Bacteroidia	Flavobacteria	Blattabacteri	Blattabacteri	cuenoti
ASV1204	Bacteria	Proteobacter	Gammaprote	Enterobacter	Yersiniaceae	Yersinia	
ASV1205	Bacteria	Proteobacter	Gammaprote	Enterobacter	Yersiniaceae	Yersinia	
ASV1206	Bacteria	Proteobacter	Gammaprote	Enterobacter	Yersiniaceae	Yersinia	
ASV1207	Bacteria	Proteobacter	Alphaproteol	Rickettsiales	Mitochondria		
ASV1208	Bacteria	Bacteroidota	Bacteroidia	Sphingobacte	Sphingobacte	Pedobacter	
ASV1209	Bacteria	Firmicutes	Bacilli	Staphylococc	Staphylococc	Staphylococcus	
ASV1210	Bacteria	Firmicutes	Negativicutes	Veillonellales	Veillonellacea	Veillonella	
ASV1211	Bacteria	Firmicutes	Bacilli	Lactobacillale	Lactobacillaceae		
ASV1212	Bacteria	Proteobacter	Gammaprote	Pseudomona	Pseudomona	Pseudomonas	
ASV1213	Bacteria	Firmicutes	Bacilli	Bacillales	Bacillaceae	Bacillus	phage
ASV1214	Bacteria	Proteobacter	Gammaprote	Enterobacter	Enterobacteriaceae		
ASV1215	Bacteria						
ASV1216	Bacteria	Proteobacter	Gammaprote	Pseudomona	Pseudomona	Pseudomonas	
ASV1217	Bacteria						
ASV1218	Bacteria						
ASV1219	Bacteria	Firmicutes	Clostridia	Peptostrepto	Family XI	Fenollaria	massiliensis
ASV1220	Bacteria						
ASV1221	Bacteria	Proteobacter	Gammaprote	Pseudomona	Moraxellacea	Acinetobacte	antiviralis
ASV1222	Bacteria	Proteobacter	Gammaprote	Enterobacter	Yersiniaceae	Yersinia	
ASV1223	Bacteria	Bacteroidota	Bacteroidia	Chitinophaga	Chitinophaga	Rurimicrobium	
ASV1224	Bacteria	Proteobacter	Gammaprote	Enterobacter	Yersiniaceae	Yersinia	pekkanenii
ASV1225	Bacteria	Proteobacter	Gammaprote	Pseudomona	Pseudomona	Pseudomonas	
ASV1226	Bacteria	Patescibacter	Saccharimona	Saccharimona	Saccharimonadaceae		
ASV1227	Bacteria						
ASV1228	Bacteria	Firmicutes	Bacilli	Lactobacillale	Listeriaceae	Listeria	
ASV1229	Bacteria	Firmicutes	Bacilli	Staphylococc	Staphylococc	Staphylococcus	
ASV1230	Bacteria	Proteobacter	Gammaprote	Enterobacter	Enterobacteriaceae		
ASV1231	Bacteria	Firmicutes	Bacilli	Staphylococc	Staphylococc	Staphylococcus	
ASV1232	Bacteria	Firmicutes	Bacilli	Lactobacillale	Streptococca	Streptococcu	lactarius
ASV1233	Bacteria	Acidobacteri	Blastocatellia	Blastocatellal	Blastocatella	Blastocatella	fastidiosa
ASV1234	Bacteria	Bacteroidota	Bacteroidia	Flavobacteria	Blattabacteri	Blattabacteri	cuenoti
ASV1235	Bacteria	Proteobacter	Gammaprote	Burkholderial	Comamonadaceae		
ASV1236	Bacteria	Firmicutes	Bacilli	Lactobacillale	Listeriaceae	Listeria	marthii
ASV1237	Bacteria	Firmicutes	Bacilli	Lactobacillales			
ASV1238							
ASV1239	Bacteria						
ASV1240	Bacteria	Proteobacter	Gammaprote	Pseudomona	Moraxellacea	Acinetobacter	
ASV1241	Bacteria	Bacteroidota	Bacteroidia	Flavobacteria	Weeksellacea	Elizabethking	bruuniana
ASV1242	Bacteria	Proteobacter	Gammaprote	Enterobacter	Aeromonada	Aeromonas	encheleia
ASV1243	Bacteria						
ASV1244	Bacteria	Proteobacter	Gammaprote	Enterobacter	Aeromonada	Aeromonas	
ASV1245	Bacteria	Proteobacter	Alphaproteol	Reyranellales	Reyranellacea	Reyranella	
ASV1246	Bacteria	Proteobacter	Gammaprote	Enterobacter	Yersiniaceae	Yersinia	
ASV1247	Bacteria	Firmicutes	Bacilli	Lactobacillale	Streptococca	Streptococcus	
ASV1248	Bacteria	Actinobacteri	Actinobacteri	Propionibacte	Propionibacte	Cutibacteriun	namnetense
ASV1249	Bacteria	Proteobacter	Gammaprote	Enterobacter	Enterobacteriaceae		

ASV1250	Bacteria	Firmicutes	Bacilli	Lactobacillale	Listeriaceae	Listeria	seeligeri
ASV1251	Bacteria	Firmicutes	Bacilli	Lactobacillale	Lactobacillaceae		
ASV1252	Bacteria	Firmicutes	Bacilli	Staphylococc	Staphylococc	Staphylococcus	
ASV1253	Bacteria	Proteobacter	Gammaproteobacteria				
ASV1254	Bacteria						
ASV1255	Bacteria	Firmicutes	Bacilli	Lactobacillale	Listeriaceae	Listeria	marthii
ASV1256	Bacteria	Firmicutes	Bacilli	Staphylococc	Staphylococc	Staphylococc	devriesei
ASV1257	Bacteria	Firmicutes	Bacilli	Lactobacillale	Enterococcac	Enterococcus	
ASV1258	Bacteria						
ASV1259	Bacteria	Actinobacteri	Actinobacteri	Corynebacter	Corynebacter	Corynebacterium	
ASV1260	Bacteria	Proteobacter	Gammaprote	Pseudomona	Pseudomona	Pseudomonas	
ASV1261	Bacteria	Firmicutes	Negativicutes	Veillonellales	Veillonellacea	Veillonella	dispar
ASV1262	Bacteria	Bacteroidota	Bacteroidia	Flavobacteria	Weeksellaceae		
ASV1263	Bacteria	Proteobacter	Gammaprote	Pseudomona	Pseudomona	Pseudomonas	
ASV1264	Bacteria						
ASV1265	Bacteria						
ASV1266	Bacteria	Proteobacter	Gammaprote	Enterobacter	Yersiniaceae		
ASV1267	Bacteria	Proteobacter	Gammaprote	Enterobacter	Yersiniaceae		
ASV1268	Bacteria	Bacteroidota	Bacteroidia	Flavobacteria	Weeksellacea	Chryseobacterium	
ASV1269	Bacteria	Proteobacter	Gammaprote	Burkholderial	Comamonada	Variovorax	paradoxus
ASV1270	Bacteria	Proteobacter	Gammaprote	Enterobacter	Yersiniaceae		
ASV1271	Bacteria						
ASV1272	Bacteria	Proteobacter	Alphaproteol	Rhizobiales	Rhizobiales Ir	Alsobacter	
ASV1273	Bacteria						
ASV1274	Bacteria	Proteobacter	Gammaprote	Enterobacter	Enterobacter	Cedecea	
ASV1275	Bacteria						
ASV1276	Bacteria	Proteobacter	Gammaprote	Enterobacter	Enterobacter	Enterobacter	
ASV1277	Bacteria	Proteobacter	Gammaprote	Pseudomona	Moraxellaceae		
ASV1278	Bacteria	Proteobacter	Gammaprote	Enterobacterales			
ASV1279	Bacteria	Firmicutes	Bacilli	Staphylococc	Staphylococc	Staphylococcus	
ASV1280	Bacteria	Bacteroidota	Bacteroidia	Bacteroidales	Dysgonomon	Petrimonas	sulfuriphila
ASV1281	Bacteria	Firmicutes	Bacilli	Bacillales	Bacillaceae	Bacillus	
ASV1282	Bacteria	Firmicutes	Bacilli	Lactobacillale	Lactobacillac	Lactobacillus	iners
ASV1283	Bacteria						
ASV1284	Bacteria	Bacteroidota	Bacteroidia	Flavobacteria	Weeksellacea	Elizabethkingia	
ASV1285	Bacteria	Proteobacter	Alphaproteol	Acetobactera	Acetobactera	Gluconobacter	
ASV1286	Bacteria	Proteobacter	Gammaprote	Enterobacterales			
ASV1287	Bacteria	Actinobacteri	Actinobacteri	Corynebacter	Corynebacter	Corynebacter	pilbarense
ASV1288	Bacteria	Actinobacteri	Actinobacteri	Micrococcales			
ASV1289	Bacteria	Proteobacter	Alphaproteol	Rhizobiales	Xanthobacteraceae		
ASV1290	Bacteria	Firmicutes	Bacilli	Lactobacillale	Enterococcac	Enterococcus	
ASV1291	Bacteria	Actinobacteri	Actinobacteria				
ASV1292	Bacteria	Firmicutes	Clostridia	Peptostrepto	Family XI	Peptoniphilus	
ASV1293	Bacteria	Firmicutes	Negativicutes	Veillonellales	Veillonellacea	Veillonella	
ASV1294	Bacteria	Firmicutes	Bacilli	Bacillales	Bacillaceae	Bacillus	idriensis
ASV1295	Bacteria						
ASV1296	Bacteria	Firmicutes	Bacilli	Bacillales	Bacillaceae	Anoxybacillus	
ASV1297	Bacteria	Firmicutes	Bacilli	Bacillales	Bacillaceae	Anoxybacillus	
ASV1298	Bacteria						
ASV1299	Bacteria						

ASV1300	Bacteria	Proteobacter	Gammaprote	Enterobacter	Enterobacteriaceae		
ASV1301	Bacteria	Proteobacter	Gammaprote	Enterobacterales			
ASV1302	Bacteria	Firmicutes	Bacilli	Lactobacillale	Listeriaceae	Listeria	seeligeri
ASV1303	Bacteria	Proteobacter	Alphaproteol	Rickettsiales	Mitochondria		
ASV1304	Bacteria	Proteobacter	Gammaprote	Pseudomona	Pseudomona	Pseudomona	phage
ASV1305	Bacteria						
ASV1306	Bacteria						
ASV1307	Bacteria	Proteobacter	Gammaprote	Burkholderial	Comamonadaceae		
ASV1308	Bacteria	Firmicutes	Bacilli	Staphylococc	Staphylococc	Staphylococcus	
ASV1309	Bacteria	Proteobacter	Gammaprote	Enterobacter	Enterobacteriaceae		
ASV1310	Bacteria	Bacteroidota	Bacteroidia	Flavobacteria	Blattabacteri	Blattabacteri	cuenoti
ASV1311	Bacteria	Proteobacter	Gammaprote	Burkholderial	Oxalobactera	Herbaspirillu	huttiense
ASV1312	Bacteria						
ASV1313	Bacteria	Proteobacter	Gammaprote	Xanthomona	Rhodanobact	Dyella	
ASV1314	Bacteria						
ASV1315	Bacteria						
ASV1316							
ASV1317	Bacteria						
ASV1318	Bacteria	Proteobacter	Gammaprote	Enterobacter	Yersiniaceae		
ASV1319	Bacteria						
ASV1320	Bacteria						
ASV1321	Bacteria						
ASV1322	Bacteria	Firmicutes	Bacilli	Lactobacillale	Lactobacillac	Limosilactobacillus	
ASV1323	Bacteria	Firmicutes	Bacilli	Bacillales	Bacillaceae	Bacillus	
ASV1324	Bacteria	Firmicutes	Bacilli	Staphylococc	Staphylococc	Staphylococcus	
ASV1325	Bacteria	Proteobacter	Gammaprote	Enterobacter	Morganellace	Morganella	morganii
ASV1326	Bacteria	Firmicutes	Bacilli	Lactobacillale	Enterococcac	Enterococcus	
ASV1327	Bacteria	Firmicutes	Bacilli	Bacillales	Bacillaceae	Bacillus	
ASV1328	Bacteria	Firmicutes	Bacilli				
ASV1329	Bacteria	Proteobacter	Gammaprote	Pseudomona	Pseudomona	Pseudomona	psychrophila
ASV1330	Bacteria	Proteobacter	Gammaprote	Pseudomona	Pseudomona	Pseudomona	psychrophila

m-Pararhizobium-Rhizobium

m-Pararhizobium-Rhizobium

m-Pararhizobium-Rhizobium
m-Pararhizobium-Rhizobium

m-Pararhizobium-Rhizobium

m-Pararhizobium-Rhizobium

m-Pararhizobium-Rhizobium

m-Pararhizobium-Rhizobium

m-Pararhizobium-Rhizobium

m-Pararhizobium-Rhizobium

m-Pararhizobium-Rhizobium

m-Pararhizobium-Rhizobium

m-Pararhizobium-Rhizobium

m-Pararhizobium-Rhizobium
